# Glio‐ and neuro‐protection by prosaposin is mediated by orphan G‐protein coupled receptors GPR37L1 and GPR37

**DOI:** 10.1002/glia.23480

**Published:** 2018-09-27

**Authors:** Beihui Liu, Valentina Mosienko, Barbara Vaccari Cardoso, Daria Prokudina, Mathew Huentelman, Anja G. Teschemacher, Sergey Kasparov

**Affiliations:** ^1^ Department of Physiology, Pharmacology, and Neuroscience University of Bristol Bristol United Kingdom; ^2^ Translational Genomics Research Institute (TGen) Phoenix Arizona; ^3^ Baltic Federal University Kaliningrad Russia Federation

**Keywords:** astrocyte, astroprotection, cAMP, GPR37, GPR37L1, neuroprotection, orphan receptors, PKA, prosaptide, Saposin C

## Abstract

Discovery of neuroprotective pathways is one of the major priorities for neuroscience. Astrocytes are natural neuroprotectors and it is likely that brain resilience can be enhanced by mobilizing their protective potential. Among G‐protein coupled receptors expressed by astrocytes, two highly related receptors, GPR37L1 and GPR37, are of particular interest. Previous studies suggested that these receptors are activated by a peptide Saposin C and its neuroactive fragments (prosaptide TX14(A)), which were demonstrated to be neuroprotective in various animal models by several groups. However, pairing of Saposin C or prosaptides with GPR37L1/GPR37 has been challenged and presently GPR37L1/GPR37 have regained their orphan status. Here, we demonstrate that in their natural habitat, astrocytes, these receptors mediate a range of effects of TX14(A), including protection from oxidative stress. The Saposin C/GPR37L1/GPR37 pathway is also involved in the neuroprotective effect of astrocytes on neurons subjected to oxidative stress. The action of TX14(A) is at least partially mediated by Gi‐proteins and the cAMP‐PKA axis. On the other hand, when recombinant GPR37L1 or GPR37 are expressed in HEK293 cells, they are not functional and do not respond to TX14(A), which explains unsuccessful attempts to confirm the ligand‐receptor pairing. Therefore, this study identifies GPR37L1/GPR37 as the receptors for TX14(A), and, by extension of Saposin C, and paves the way for the development of neuroprotective therapeutics acting via these receptors.

A video abstract of this article can be found at: https://www.youtube.com/watch?v=qTn13My9Sz8

## 1 INTRODUCTION

Any new target for effective neuroprotective therapy must be actively explored as it may have major medical and societal impacts. Orphan G‐protein‐coupled receptors (GPCRs) are particularly attractive because they are the most plausible targets for modern small molecule drugs, but this approach critically depends on identification of their endogenous agonists. The search for druggable targets in the brain conventionally focused on neurons, but astrocytes as natural neuroprotectors represent particularly attractive drug targets. Their neuroprotective mechanisms are numerous and include uptake of glutamate to prevent neurotoxicity, regulation of extracellular ions and pH, provision of antioxidative molecules (e.g., glutathione) and trophic factors, control of micro‐circulation, etc. (Liu, Teschemacher, & Kasparov, 2017).

In 1994, the peptide prosaposin (PSAP) and its fragment Saposin C (Sap C) were identified as neurotrophic factors using the neuroblastoma NS20 line and specific binding of radio‐labeled Sap C with a *K*_d_ of 19 pM was demonstrated (O'Brien, Carson, Seo, Hiraiwa, & Kishimoto, 1994). Soon after it was shown that chronic icv infusion of recombinant PSAP almost completely prevented ischemia‐induced learning deficits and neuronal loss in gerbils (Sano et al., 1994) and the existence of a GPCR for the neuroprotective part of Sap C was thus postulated (Hiraiwa, Campana, Martin, & O'Brien, 1997). The experimental usefulness of PSAP and Sap C is limited by their length but, luckily, the neuroactive part is rather short and can be mimicked by peptides known as prosaptides, of which the most studied is prosaptide TX14(A). The sequence of TX14(A) is highly evolutionarily conserved (Supporting Information Figure S1). Although neuroprotective effects of PSAP fragments were demonstrated in several models in vitro and in vivo (Campana et al., 1998; Gao et al., 2016; Hozumi et al., 1999; Otero, Conrad, & O'Brien, 1999), the underlying mechanism remained unclear until 2013, when the two closely related orphan receptors GPR37L1 and GPR37 (Leng, Gu, Simerly, & Spindel, 1999) were proposed to mediate the actions of PSAP and its mimetics (Meyer, Giddens, Schaefer, & Hall, 2013).

GPR37L1 is highly expressed by astrocytes, which also express low levels of GPR37 (Supporting Information Figures S2 and S3; Jolly et al., 2017; Marazziti et al., 2007; Smith, 2015; Zhang et al., 2014). GPR37 is highly expressed in dopaminergic neurons and early work focused on the idea of GPR37 being involved in Parkinson's disease (Cantuti‐Castelvetri et al., 2007; Imai et al., 2001; Marazziti et al., 2007). The importance of GPR37L1 for brain function has been recently demonstrated in humans. A point mutation in GPR37L1 leads to a severe neurological phenotype which includes intractable epilepsy, lethal in some of the affected individuals (Giddens et al., 2017). GPR37L1‐ and especially double GPR37L1/GPR37‐knockout mice were highly susceptible to seizures (Giddens et al., 2017). Moreover, the deletion of GPR37L1 drastically increased the neuronal loss after an ischemic stroke (Jolly et al., 2017). These and other findings underscore the importance of GPR37L1 for brain health.

However, the pairing of GPR37L1/GPR37 with PSAP and TX14(A) (Meyer et al., 2013) was later challenged. It was reported that these receptors are highly constitutively active and couple via Gs proteins, rather than the Gi pathway as originally reported (Meyer et al., 2013). Moreover, on the background of their high constitutive activity, TX14(A) was ineffective (Coleman et al., 2016; Giddens et al., 2017; Ngo et al., 2017). Regulation of this constitutive activity was suggested to occur via cleavage of the extracellular part of GPR37L1 (Coleman et al., 2016; Mattila, Tuusa, & Petaja‐Repo, 2016). These reports reinforced the skepticism based on the failure of TX14(A) to activate GPR37L1/GPR37 using the DiscoverX orphan receptor‐screening panel (Smith, 2015; Southern et al., 2013). Importantly, all studies reporting high constitutive activity of GPR37L1 and GPR37 and lack of TX14(A) agonism relied on expression of recombinant GPR37L1 in either HEK293 or CHO cells (Giddens et al., 2017; Ngo et al., 2017; Southern et al., 2013) or yeast (Coleman et al., 2016).

Thus, the nature of the endogenous agonist of GPR37L1 and GPR37 is currently elusive.

High constitutive activity of GPR37L1/GPR37 should lead to persistent production of copious amounts of cAMP. However, this has never been noticed in astrocytes where GPR37L1 is particularly abundant (Supporting Information Figures S2 and S3). To the contrary, astrocytes vigorously respond to stimuli which increase cAMP production (such as agonists of Gs‐coupled receptors or low concentrations of forskolin), indicating that their resting levels of cAMP are not anywhere near saturation (see, for example, Clark and Perkins, 1971; Goldman and Chiu, 1984; Tardy et al., 1981).

We hypothesized that coupling of GPR37L1/GPR37 in transiently transfected cell lines does not reveal their true physiological signaling and reevaluated them in their natural habitat, the astrocytes. Our findings demonstrate that PSAP is, indeed, the natural ligand of GPR37L1/GPR37 and pave the way for development of neuroprotective drugs based on this signaling system.

## 2 MATERIALS AND METHODS

### 2.1 Primary cultures of astrocytes and cortical neurons

Experiments were performed in accordance with the UK Animals (Scientific Procedures) Act, 1986, and were approved by the University of Bristol ethics committee.

#### 2.1.1 Astrocytes

Primary cultures of astrocytes were prepared from the cerebral cortices, cerebellum, and brainstem from Wistar rat pups (P2) following protocols described previously (1–3). Briefly, the brains of Wistar P2 pups were dissected out, crudely cross‐chopped and bathed in a solution containing HBSS, DNase I (0.04 mg/mL), trypsin from bovine pancreas (0.25 mg/mL) and BSA (3 mg/mL). The preparation was agitated at 37 °C for 15 min. Trypsinization of the brain tissue was terminated by the addition of equal volumes of culture media comprised of DMEM, 10% heat‐inactivated FBS, 100 U/mL penicillin, and 0.1 mg/mL streptomycin and then centrifuged at 2000 rpm, at room temperature (RT) for 10 min. The supernatant was aspirated, and the remaining pellet was resuspended in 15 mL HBSS containing BSA (3 mg/mL) and DNase I (0.04 mg/mL) and triturated gently. After the cell debris had settled, the cell suspension was filtered through a 40‐μm cell strainer (BD Falcon, BD Biosciences, Franklin Lakes, NJ) and cells were collected after centrifugation. Cells were seeded in a T75 flask containing culture media (see above) and maintained at 37 °C with 5% CO_2_. Once the cultures reached confluence and 1 week later, the flasks were mildly shaken overnight to remove microglia and oligodendrocytes. When astrocytes were seeded for experiments, media was changed to DMEM supplemented with 5% FBS instead of 10% FBS. This is to reduce the content of PSAP in the culture media hence make PSAP depletion easier to achieve. Note that there was no difference in cell growth in the media containing either 5% or 10% FBS.

#### 2.1.2 Neurons

Cerebral cortices were dissected out from a litter (8–12) of Wistar rat embryos on gestation day 18 (E18) and collected in dissection saline (HBSS, 25.6 mM glucose, 10 mM MgCl_2_, 1 mM Hepes, 1 mM kynurenic acid, 0.005% phenol red, 100 U/mL penicillin, and 0.1 mg/mL streptomycin). Meninges were removed, and tissues were chopped into pieces <1 mm^3^ and dissociated in 0.25% Trypsin in dissection saline in the presence of 3 mg/mL BSA at 37 °C for 15 min. An equal volume of plating media (Neurobasal A with 5% horse serum, 2% B27, 400 nM l‐glutamine, 100 U/mL penicillin, and 0.1 mg/mL streptomycin) was added to terminate the dissociation. Cells were pelleted at 2000 rpm for 5 min at room temperature, resuspended in plating media, and triturated gently. The cell suspension was diluted appropriately and passed through a 40‐μM cell strainer. Approximately 1 × 10^5^ cells per well were plated on poly‐d‐lysine‐coated glass cover slips in 24‐well plates. Two hours later, the plating media was replaced with feeding media (Neurobasal A with 2% B27, 800 nM l‐glutamine, 20 U/mL penicillin, and 20 μg/mL streptomycin). On day 5, half of the media was replaced with feeding media in which glutamine was replaced with 4 μM Glutamax. The antimitotic cytosine β‐d‐arabinofuranoside (10 μM) was added to control glial contamination. Neurons were used for experiments 10 days later.

##### Neuron/astrocyte co‐cultures

Neurons were prepared (see above) and plated at 1 × 10^5^ cells per well on poly‐d‐lysine‐coated glass coverslips in 24‐well plates. Astrocyte inserts were prepared by plating astrocytes on poly‐d‐lysine‐coated cell culture inserts with 1 μm diameter pores (Greiner Bio‐One, Monroe, NC) in the same serum‐free media as used for neurons. Astrocyte inserts were introduced into neuronal cultures as required. The separation between both cell types allowed secreted molecules to freely diffuse while preventing direct astrocyte‐to‐neuron contact.

### 2.2 Real‐time PCR on primary cultured and acutely isolated astrocytes

To verify that the expression of GPR37L1 and GPR37 in our cultured astrocytes is not a result of in vitro conditions, we performed acute vibro‐isolation of cortical astrocytes from P12 rats using a method recently described by Lalo and Pankratov (2017). Approximately 50 single astrocytes were manually collected from the bottom of a small Petri dish into a sterile test tube. Power SYBR Green Cells‐to‐Ct Kit (Ambion Inc., Austin, TX) was used to reverse transcribe directly from cultured cell lysates, without isolating RNA. The resulting cDNA samples were then analyzed using QuantiTect SYBR green PCR kit (Qiagen, Hilden, Germany) on DNA Engine OPTICON 2 continuous fluorescence detector, following the manufacturer's protocol. β‐Actin was used as a reference house‐keeping gene. All primers were designed to span at least one intron and to produce products of ~100 bp and prevalidated for their efficiency. Products of PCR reaction were resolved on agarose gel to confirm their sizes (Supporting Information Figure S3). Sequences of the primers are: β‐Actin forward: CTAAGGCCAACCGTGAAAAG; reverse: GGCATACAGGGACAACACAG; GPR37L1 forward: ATGTTTCTTGCCGAGCAGTG; GPRF37L1 reverse: CCACATGGAATCGGTCTATG; GPR37 forward: TCCATGAGTTGACCAAGAAG; GPR37 reverse: CTATGCACAGTGCACATAAG; GFAP forward: GAGAGGAAGGTTGAGTCGCT; GFAP reverse: CACGTGGACCTGCTGCTG.

### 2.3 Western blotting

For verification of GPR37L1/37 knock‐down with adenoviral vectors (AVV), transduced astrocytes were harvested and placed on ice and washed with ice‐cold phosphate‐buffered saline (PBS). The membrane proteins were then extracted using the Mem‐PER eukaryotic membrane protein extraction reagent kit (PIERCE) and purified with SDS‐PAGE sample preparation kit (PIERCE, Appleton, WI). After quantification with BCA protein assay kit (PIERCE), 20 μg of membrane protein per lane were fractionated on a 4–12% Bis–Tris gel (NuPage 4–12% Bis–Tris Gel, Life Technologies, Carlsbad, CA), and transferred to a polyvinylidene difluoride (PVDF) membrane (Millipore, Burlington, MA). After blocking with 5% nonfat dry milk (NFDM) in Tris‐buffered saline with 0.1% Tween‐20 (TBST) buffer for 45 min at RT, the PVDF membrane was cut into two parts at 100 kDa size level. The part of the membrane containing small sized proteins was incubated with primary antibody to GPR37L1 (1:1000 dilution) or GPR37 (1:1000 dilution) in 3% NFDM‐TBST at 4 °C overnight, and the other part of membrane was incubated with primary antibody to pan‐cadherin (120 kDa) (1:2000 dilution) as a membrane protein loading control, in 3% NFDM‐TBST at 4 °C overnight. Following incubation with horseradish peroxidase conjugated secondary antibody (DAKO, 1:2000 dilution) for 90 min at RT, the immunoreactivities were detected with Immun‐Star Western C chemiluminescent kit (Bio‐Rad Laboratories, Hercules, CA). For the PSAP depletion assay and the proof of the existence of PSAP in serum‐supplemented culture media, we used the same protocol as described above except that 5 μL of media or elution from protein A magnetic beads was applied as the sample volume. A polyclonal rabbit anti‐PSAP antibody was employed. For Western blotting of PSAP in neuron–astrocyte co‐culture media, we changed to the Amersham ECL Plex western blotting system using a low‐fluorescent PVDF membrane (GE Healthcare, Chicago, IL) and Alexa Fluor 488 secondary antibody‐conjugated goat anti‐rabbit. Protein transfer and membrane blocking was the same as in the above protocol. Membranes were incubated with anti‐PSAP (1:500 dilution) overnight at 4 °C. Secondary antibody Alexa Fluor 488 was incubated for 1 hr in the dark at room temperature. Before imaging, the membrane was thoroughly washed. Signal was detected by scanning the membrane on a fluorescent laser scanner (Typhoon, GE Healthcare).

### 2.4 Generation of knock‐down AVV

AVV for the knock‐down experiments were based on a modified Pol II miR RNAi Expression Vector system (Invitrogen) and our previous work (Liu, Xu, Paton, & Kasparov, 2010). Three AVV were constructed, namely, AVV–CMV–EmGFP–miR155/GPR37L1, AVV–CMV–EmGFP–miR155/GPR37, and AVV–CMV–EmGFP–miR155/negative. The first two were used for knocking down GPR37L1 and GPR37 in astrocytes, respectively. The third one is a negative control, harboring a miRNA sequence that can form a hairpin structure that is processed into mature miRNA but is predicted not to target any known vertebrate gene. All three vectors were made based on BLOCK‐iT Pol II miR RNAi Expression Vector Kit with EmGFP (Invitrogen, Carlsbad, CA). This system supports chaining of miRNAs, thus ensuring co‐cistronic expression of multiple miRNAs for knock down of a single target. Three sets of two complementary single‐stranded DNA micro‐RNA sequences (targeting different regions of the same gene) for GPR37L1 and GPR37 were designed by the BLOCK‐iT RNAi Designer (Invitrogen). The complementary single‐stranded oligos were then annealed and cloned into the linearized pcDNA 6.2‐GW/EmGFP–miR vector. The pre‐miRNA expression cassettes were transferred into an adeno shuttle plasmid pXcX–Sw‐linker (Duale, Kasparov, Paton, & Teschemacher, 2005), resulting in construction of pXcX–CMV–EmGFP–miR155/GPR37L1, pXcX–CMV–EmGFP–miR155/GPR37 and pXcX–CMV–EmGFP–miR155/negative. AVV were then produced by homologous recombination of shuttle and the helper plasmid pBHG10 in HEK293 cells. The media was collected for subsequent rounds of AVV proliferation in HEK293 cells until cytopathic effects were achieved. AVV were purified using CsCl gradient protocols. Titers were established using an immunoreactivity spot assay as described previously (Duale et al., 2005).

### 2.5 PSAP depletion

To deplete PSAP from culture media (DMEM supplemented with 5% FBS), initially, 25 μL of Protein A Magnetic Beads (NEB) per 200 μL media were added and incubated for 1 hr at 4 °C. Magnetic field was applied for 30 s to pull beads to the side of the tube and the supernatant was transferred to a new tube. This step is required to remove non‐specific binding proteins. Then, 5 μL of anti‐PSAP antibody (500 μg/mL) were added to the supernatant and incubated for 1 hr at 4 °C. Protein A magnetic beads were used again to pull down the antibody‐bound PSAP. Western blot was used to verify the efficient removal of PSAP which could then be recovered from the beads (Supporting Information Figure S4).

### 2.6 cAMP assays

Two types of detection were used.

#### 2.6.1 Luminescence activity‐based GloSensor assay (Promega)

The GloSensor assay uses genetically encoded biosensor variants with cAMP binding domains fused to mutant forms of *Photinus pyralis* luciferase. The luminescence of the reporter increases directly proportionally to the amount of cAMP present. Astrocytes were seeded in 96‐well plates and transduced with an AVV bearing the CMV‐driven GLO22F at multiplicity of infection (MOI) 10. After 24 hr transduction, media was exchanged for 100 μL HEPES‐buffered HBSS (pH 7.6). Cells were then incubated with 0.731 mM beetle luciferin for 2 hr in the dark. After baseline reading, NKH477 and/or TX14(A) were added to the wells and incubated for 20 min. Luminescence measurements were obtained using a Tecan microplate reader (Infinite M200 PRO).

#### 2.6.2 FRET‐based cAMP assay

Two to three days before recordings, astrocytes were plated onto coverslips in prosaposin‐depleted media (PDM) and transduced with an AVV to express an Epac [Exchange protein directly activated by cAMP)‐based FRET sensor (kindly provided by K. Jalink, Amsterdam; Klarenbeek, Goedhart, van Batenburg, Groenewald, & Jalink, 2015; Klarenbeek & Jalink, 2014) specifically in astrocytes. PDM was exchanged daily. For recording, coverslips were placed into a chamber on a confocal microscope and continuously superfused with HEPES‐buffered solution (HBS; in mM: NaCl 137, KCl 5.4, Na_2_HPO_4_ 0.34, KH_2_PO_4_ 0.44, CaCl_2_ 1.67, MgSO_4_ 0.8, NaHCO_3_ 4.2, HEPES 10, Glucose 5.5; pH 7.4, 31.4 °C). After taking baseline readings of astrocytes in the field of view, cells were exposed to 5 min of NKH477 (0.5 μM), alone or in combination with TX14(A) (100 nM). Fluctuations in cAMP were monitored through CFP/YFP (465–500 nm/515–595 nm bands) emission ratios upon 458 nm light excitation. Images were acquired every 4 s. All FRET ratios were normalized to baseline.

### 2.7 PRESTO‐Tango β‐arrestin‐recruitment assay

To measure receptor activation, the PRESTO‐Tango β‐arrestin‐recruitment assay was performed as previously described, with modifications (Kroeze et al., 2015). HTLA cells, a HEK293 cell line stably expressing a tTA‐dependent luciferase reporter and a β‐arrestin2‐TEV fusion gene, were used. The cells were maintained in DMEM media supplemented with 10% FBS, 100 U/mL penicillin and 100 μg/mL streptomycin, 2 μg/mL puromycin, and 100 μg/mL hygromycin B. For transfection, HTLA cells were plated in 96‐well white polystyrene plates (Greiner Bio‐One, Monroe, NC) in DMEM media supplemented with 10% FBS, 100 U/mL penicillin, and 100 μg/mL streptomycin at a density of 4 × 10^5^ cells/mL. For measuring activation of GPR37 and GPR37L1 by TX14(A) stimulation, PDM was used instead. After 16 hr, cells were transfected with the plasmid containing the GPR37 or GPR37L1 ORF (Addgene, Cambridge, MA) using Trans‐IT 293 (Mirus Bio, Madison, WI) according to manufacturer's protocol. On the next day, drugs were added in assay buffer (20 mM HEPES in HBSS, pH 7.4) and left to incubate for 24 hr. Solutions were aspirated, and 80 μL per well of Bright‐Glo solution (Promega, Madison, WI) diluted 20‐fold in assay buffer was added to each well in the dark. Following 20 min of incubation, luminescence measurements were obtained using a Tecan microplate reader (Infinite M200 PRO, Tecan Trading AG, Männedorf, Switzerland).

### 2.8 Scratch wound assay

A wound recovery assay was carried out to analyze the migration of astrocytes using the IncuCyte system (Essen BioScience Inc., Ann Arbor, MI). Primary astrocytes were seeded in ImageLock 96‐well plates (4,379, Essen BioScience) at a density of 4 × 10^4^ per well. After 24 hr, standardized and reproducible (700–800 μm wide) scratch “wounds” were created in all wells using a dedicated device. Cultures were exposed to different testing conditions, for example, stressors, and were maintained and imaged at hourly intervals up to 72 hr.

Cell density was measured in the scratch area and compared to undisrupted adjacent monolayer. Relative wound density (%), a measure of wound recovery, was calculated using the formula: Relative wound density (%) = (Density of wound region at certain time point – Initial density of wound region)/(Density of intact cell region at certain time point − Initial density of wound) × 100%.

### 2.9 DAPI staining

Astrocytes were seeded in 96‐well plate (1 × 10^4^ per well) and fixed in 4% paraformaldehyde for 5 min, washed in PBS three times for 5 min each time before and after incubating with 1 μg/mL DAPI for 10 min. Round and whole nuclei in nine fields of view per well were counted using ImageJ (NIH, Bethesda, MD).

### 2.10 BrdU incorporation assay

To detect cell division, astrocytes were seeded in a 96‐well plate (4 × 10^3^ per well) in media supplemented with 5% FBS or PDM and cultured for 1 day. BrdU was added to the wells at a final concentration of 10 μM. After 24 hr, cells were fixed with 4% paraformaldehyde in PBS (pH 7.4) for 10 min at room temperature and permeabilized with 0.1–0.25% Triton X‐100 in PBS. Cells were then incubated with 1 M HCl for 30 min, followed by primary antibody incubation with mouse monoclonal anti‐BrdU antibody (1:100) containing 2.5% goat serum at 4 °C overnight. Before imaging, cells were incubated with Alexa Fluor 488‐conjugated goat anti‐mouse secondary antibody for 30 min at room temperature.

### 2.11 Lactate dehydrogenase release assay

Lactate dehydrogenase (LDH) release from damaged cells was assessed colorimetrically with LDH Cytotoxicity Assay Kit (Pierce) according to the manufacturer's instructions. Activity is proportional to colorimetric reduction of tetrazolium salt measured at 490 nm. Cytotoxicity was normalized to maximal LDH activity as released from cells acutely exposed to Triton X‐100, and calculated using the formula: % Cytotoxicity = (Compound‐treated LDH activity – Spontaneous LDH activity)/(Maximum LDH activity – Spontaneous LDH activity) × 100%.

To determine the protective effect of prosaptide TX14(A) against oxidative stress on primary astrocytes, cells were seeded in triplicates in 96‐well plates (4 × 10^4^ per well) in PDM and transduced with AVV–CMV–EmGFP–miR155/negative (labeled as miRNA‐negative in the figures), or a mixture of AVV–CMV–EmGFP–miR155/GPR37L1 and AVV–CMV–EmGFP–miR155/GPR37 (molar ratio 3:1, labeled as miRNA‐GPR37L1/GPR37 in figures). 24 hr later, the media was replaced by fresh PDM and cells were treated with H_2_O_2_ (250 μM), staurosporine (200 nM), or rotenone (100 μM) for 5 hr in the presence or absence of TX14(A). The media was then replaced by fresh PDM with or without TX14(A). Cells were incubated for a further 24 hr before carrying out the LDH reaction on 50 μL of media.

For the protective effect of astrocytes on stressed neurons in the co‐culture system, astrocytes or neurons were transduced with AVV at MOI 10. For astrocytes, cells were transduced at the time of plating on cell culture inserts. For neurons, cells were transduced on day 3 after preparation and then plated. After 24 hr, media were replaced. Cells were cultured for 7 more days before they were treated with H_2_O_2_ (250 μM, 1 hr), rotenone (50 μM, 2 hr), or staurosporine (100 nM, 2 hr). Stressors were then removed and neurons were incubated with or without astrocytes inserts. After 24 hr, the LDH reaction was carried out using 50 μL of media. Cytoprotection was calculated using the formula: % Cytoprotection = % cytotoxicity in the control condition − % cytotoxicity in the experimental condition.

### 2.12 Reagents

Cell culture and cell‐based assays related: Beetle luciferin potassium salt (E1602, Promega); B27 (17,504,044, Life Technologies, Carlsbad, CA); Bovine serum albumin (BSA) fraction V (A3294, Sigma, Kawasaki, Kanagawa Prefecture, Japan); BrdU (AB142567, Abcam); Bright‐Glo reagent (E2610, Promega); cytosine β‐d‐arabinofuranoside (C1768, Sigma); DNase I (D5025, Sigma); DAPI (D9542, Sigma); Dulbecco's modified eagle medium (DMEM; 61,965, Life Technologies); fetal bovine serum (FBS, 10082147, Life Technologies); GlutaMax (35,050,038, Life Technologies); l‐Glutamine (2,503,008, Life Technologies); Hank's balanced salt solution (HBSS; 14175‐129, Invitrogen); HEPES (H3375, Sigma); horse serum (H1138, Sigma); hygromycin B (H3274, Sigma); kynurenic acid (K3375, Sigma); neurobasal‐A media (10,888,022, Life Technologies); penicillin/streptomycin (15140‐122, Life Technologies); poly‐d‐lysine (A‐003‐E, Millipore); protein A magnetic beads (NEB, S1425S); puromycin (P8833, Sigma); Triton X‐100 (T8787, Sigma); Trypsin (type III, bovine fraction; T9935, Sigma). Ambion Power SYBR Green Cells to Ct kit (4402953) was used to verify GPR37L1 and GPR37 expression in cultured and acutely isolated astrocytes.

Antibodies: Alexa Fluor 488‐conjugated goat anti‐rabbit secondary antibody (R37116, Life Technologies); Alexa Fluor 488‐conjugated goat anti‐mouse secondary antibody (R37120, Life technologies); GPR37 L1 antibody (AB151518, Abcam, Cambridge, UK); GPR37 antibody (14820–1‐AP, Proteintech, Chicago, IL); mouse monoclonal anti‐BrdU antibody (GTX27781, GeneTex, Irvine, CA); pan‐cadherin antibody (AB6529, Abcam); rabbit anti‐PSAP antibody (AB68466, Abcam).

Drugs: 6‐BenZ‐cAMP (B009‐10, BIOLOG Life Science Institute, Bremen, Germany); 8‐pCPT‐2′‐O‐Me‐cAMP (C041‐05, BIOLOG Life Science Institute); H_2_O_2_ (H1009, Sigma); Pertussis toxin (3,097, Tocris Bioscience, Bristol, UK); NKH 477 (SC‐204130, Santa Cruz Biotechnology, Dallas, TX); Prosaptide TX14(A) (5,151, Tocris); rotenone (R8875, Sigma); Staurosporine (10,042,804, Fisher Scientific, Hampton, NH).

All other chemicals were from Sigma.

### 2.13 Statistical analysis

All data analysis was performed with GraphPad Prism 7 (GraphPad Software Inc., La Jolla, CA). One‐way ANOVA with post hoc analysis was used, unless otherwise stated. *p < .05, **p < .01, ***p < .001, ****p < .0001. Grouped data are presented as mean ± *SD*, unless otherwise stated.

Further details of statistical procedures can be found in the Supporting Information as “Supplemental Statistics.”

## 3 RESULTS

### 3.1 GPR37L1/GPR37 activation by prosaptide inhibits cAMP production in astrocytes but not in HEK293 cells

**Figure 1:**
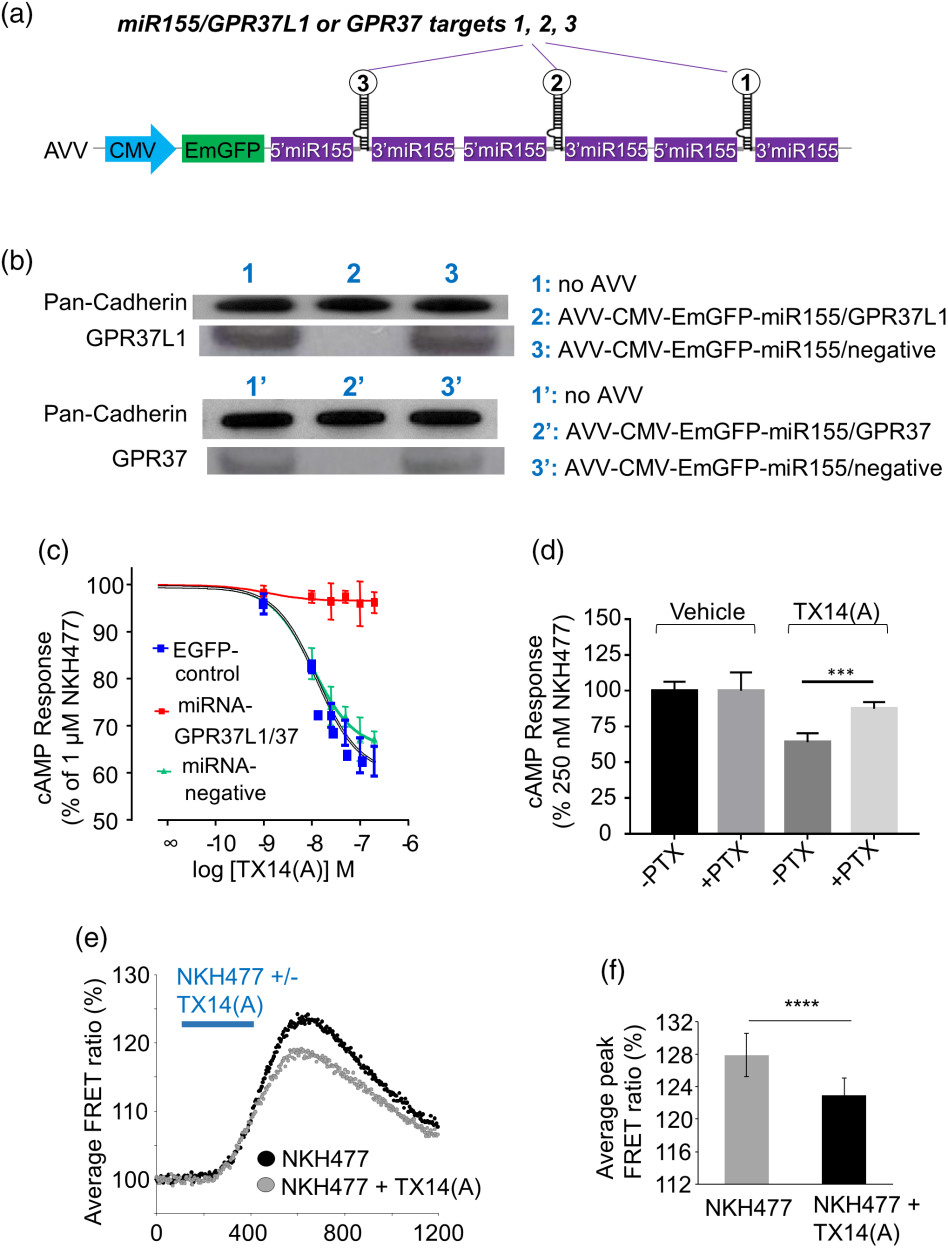
TX14(A) acting on GPR37L1/GPR37 reduces cAMP levels in astrocytes. (a) Layout of the adenoviral vectors for knock‐down of GPR37L1 and GPR37. Each vector allows co‐cistronic expression of three pre‐miRNAs targeting different regions of the target gene. AVV = human adenoviral vectors serotype 5; CMV = human cytomegalovirus promoter; EmGFP = Emerald green fluorescent protein; miR155 = flanking pre‐miRNA sequence derived from miR‐155. (b) Western blot confirms that AVV–CMV–EmGFP–miR155/GPR37L1 and AVV–CMV–EmGFP–miR155/GPR37 (MOI 10) efficiently knock‐down GPR37L1 and GPR37 in astrocytes. AVV–CMV–EmGFP–miR155/negative is a control vector with hairpin sequence relevant to no known vertebrate gene. (c) Concentration–response curves for inhibition of cAMP production by TX14(A) in astrocytes pretreated with 1 μM NKH477. Cells were transduced with AVV–CMV–Glosensor and either AVV–CMV–EmGFP (control), a mixture of AVV–CMV–EmGFP–miR155/GPR37L1 and AVV–CMV–EmGFP–miR155/GPR37 to knock‐down GPR37L1/GPR37, or with AVV–CMV–EmGFP–miR155/negative (*n* = 4, triplicates). (d) AVV–CMV–Glosensor transduced astrocytes were pretreated with PTX (20 hr, 100 ng/mL). About 100 nM TX14(A)‐induced cAMP reduction in astrocytes was PTX sensitive (*n* = 12, ***p < .001 vs. indicated group, one‐way ANOVA with Turkey's post hoc analysis). (e): Astrocytes expressing an EPAC‐based cAMP sensor were kept in PSAP‐depleted media overnight and were stimulated with NKH477 (0.5 μM) in the absence or presence of TX14(A) (100 nM). TX14(A) decreased the transient cAMP signal; average of 58 astrocytes from four experiments. (f) Pooled data from (e) shows significantly decreased FRET ratio peaks with TX14(A) (*n* = 58 = ****p < .0001, paired *t* test) [Color figure can be viewed at wileyonlinelibrary.com]

Consistent with published information, GPR37L1 is strongly expressed by cultured rat astrocytes which also express GPR37 at a lower level. We have also confirmed that both receptors are present in acutely isolated cortical astrocytes from P12 rats, consistent with various published transcriptomes (Supporting Information Figures S2 and S3). Therefore, we always targeted both receptors simultaneously. A powerful double knock‐down of GPR37L1 and GPR37 was achieved by modifying conventional micro‐RNA based cassettes to incorporate three anti‐target hairpins fused to the 3′‐end of the Emerald green protein (Figure 1a; Liu et al., 2010). These “triple‐hit” cassettes suppressed GPR37L1 and GPR37 protein expression below our detection limits whereas a negative control sequence had no impact (Figure 1b). The efficacy of the knock‐down was additionally confirmed using real‐time PCR (Supporting Information Figure S5). To ensure maximal transduction of astrocytes we used AVV which are exceptionally effective tools for these cells (Supporting Information Figure S6).

To assess intracellular cAMP changes, AVV were also used to express the Glosensor biosensor (Promega). Baseline levels of cAMP were raised ~20‐fold using a water soluble forskolin analogue, NKH477. On that background, TX14(A) concentration dependently decreased cAMP with an IC_50_ of 17.8 nM (Figure 1c). The maximal concentration of TX14(A) used (200 nM) inhibited NKH477‐mediated cAMP production by ~40% (Figure 1c). The decrease of cAMP induced by TX14(A) was pertussis toxin (PTX) sensitive (Figure 1d). Expression of the negative control miRNA vector had no effect while combined knock‐down of the GPR37L1 and GPR37 receptors completely obliterated the TX14(A)‐mediated decrease in cAMP levels (Figure 1c). In addition, we employed an EPAC‐based high affinity FRET sensor for cAMP (Klarenbeek et al., 2015) to visualize cAMP dynamics following TX14(A) application and found that TX14(A) (100 nM) significantly reduced the NKH477‐evoked rise in FRET ratio (Figure 1e,f).

**Figure 2:**
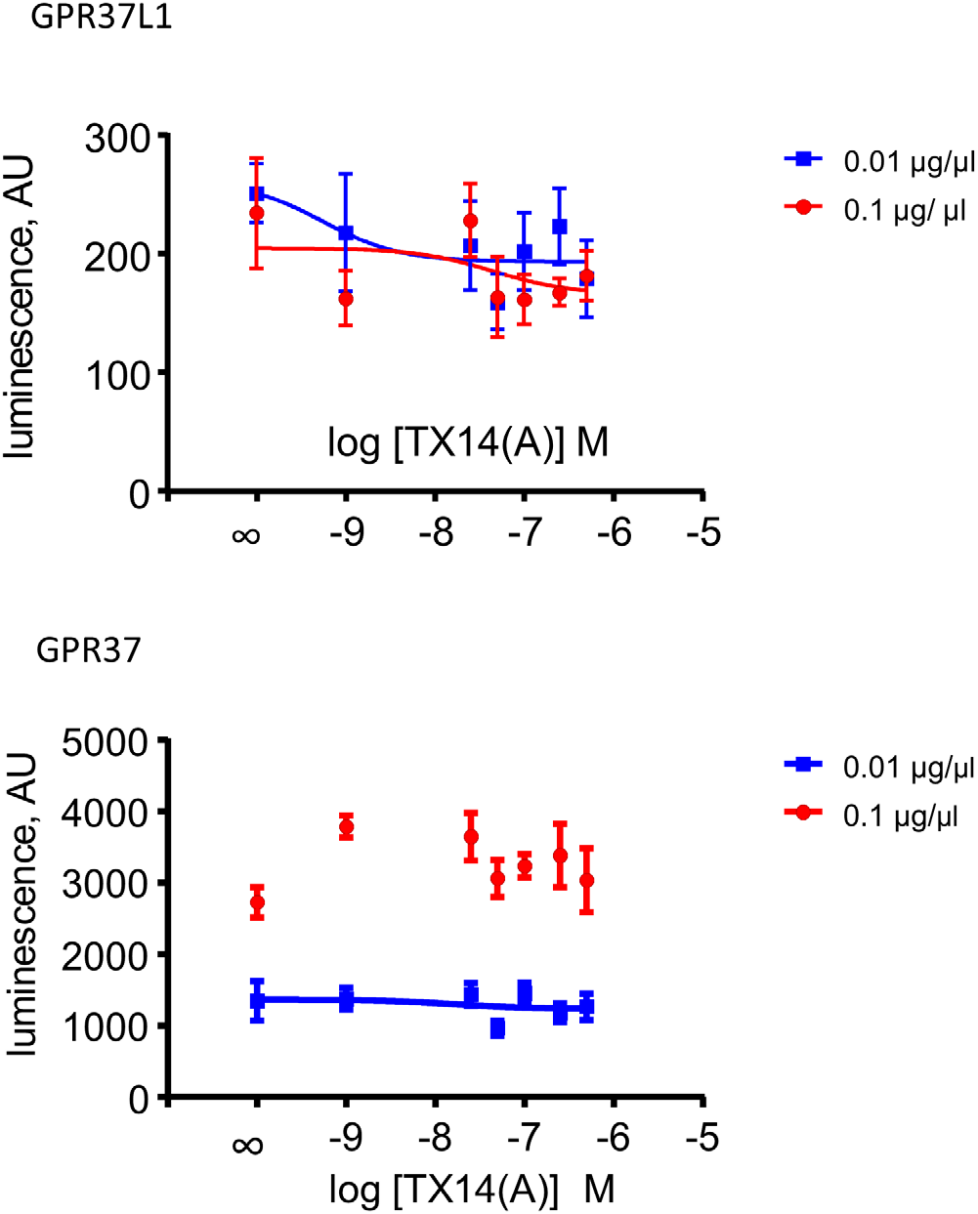
GPR37L1 and GPR37 are non‐responsive to prosaptide TX14(A) in PRESTO‐Tango assay in HEK293 cells. PRESTO‐Tango uses clones of numerous human GPCR, C‐terminally tagged with a special signaling element. These receptors need to be expressed in a specially designed clone of HEK293 cells. Agonist binding triggers receptor internalization and eventually leads to expression of luciferase and luminescence. Two concentrations of plasmid DNA were used to express the tagged receptors. GPR37 exhibits strong constitutive activity, especially when using 0.1 μg/μL. Values obtained with 0.1 μg of GPR37 DNA could not be fitted with the regression algorithm of Prism software, hence no line is shown (*n* = 6) [Color figure can be viewed at wileyonlinelibrary.com]

Several previous reports which failed to confirm the TX14(A) effect on GPR37L1 and GPR37 used transiently transfected HEK293 cells. To verify that in HEK293 cells GPR37L1 and GPR37 signaling is different to that in astrocytes (Figure 1c–f), we used the PRESTO‐Tango system (Kroeze et al., 2015). It provides GPCRs adapted for transient expression in a HEK293 line, modified to detect agonist‐induced GPCR internalization. In that assay, GPR37 was constitutively active (compared to the baseline with ADRA1; Supporting Information Figure S7) and both receptors were insensitive to prosaptide TX14(A) (Figure 2), while the α‐adrenoceptor 1a (ADRA1a) demonstrated an appropriate response (Supporting Information Figure S7). The most likely explanation for this difference is that GPR37L1 and GPR37 require some additional proteins for their correct function which are lacking in HEK293 cells. Further analysis of this issue is outside of the scope of the current study.

### 3.2 PSAP and TX14(A) acting via GPR37L1/GPR37 are essential for the motility of astrocytes

**Figure 3:**
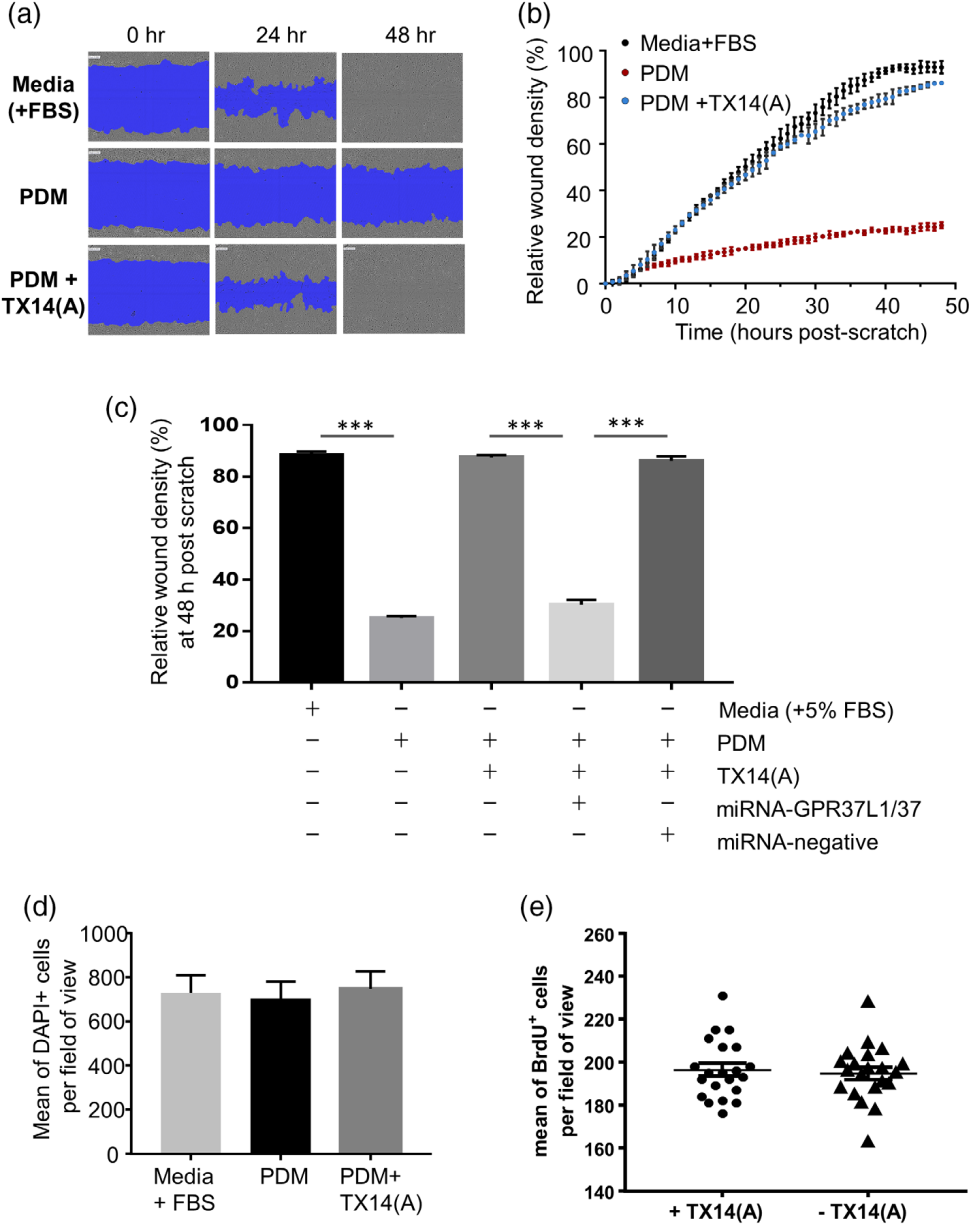
PSAP/GPR37L1/GPR37–mediated signaling is essential for migration of astrocytes in the scratch assay and the effect of PSAP is mimicked by TX14(A). (a) Astrocytes move into a wound in media containing 5% FBS and in PDM (prosaposin‐depleted media) supplemented with 100 nM TX14(A) but the movement is inhibited in absence of PSAP. Representative images at 0, 24, and 48 hr post scratch. See also Supporting Information Movies SS1–SS3. (b) Dynamics of the relative wound density under conditions shown in a (*n* = 3, triplicates). (c) Relative wound density 48 hr post scratch for astrocytes incubated in FBS‐containing media, PDM, PDM + TX14(A), PDM + TX14(A) + both knock‐down vectors and PDM + TX14(A) + knock‐down control vector *n* = 6, triplicates, ***p < .001 vs. indicated group, one‐way ANOVA with Turkey's post hoc analysis). (d): DAPI staining revealed no significant differences in the cell density between astrocytes cultured in media (+FBS), PDM and PDM supplemented with TX14(A) (100 nM; *n* = 15). (e): The addition of TX14(A) to PDM does not affect the numbers of new astrocytes based on BrdU staining (*n* = 7, triplicates) [Color figure can be viewed at wileyonlinelibrary.com]

Body fluids, such as milk, blood, and cerebrospinal fluid, contain PSAP (Kishimoto, Hiraiwa, & O'Brien, 1992). Most media used for culturing are supplemented with FBS. Unsurprisingly, PSAP was present in FBS‐supplemented media (Supporting Information Figure S4). We studied the effect of PSAP depletion, using immunoadsorption (Supporting Information Figure S4), on the motility of astrocytes in a wound scratch assay. Reproducible (700–800 μM wide) scratch wounds were created in astrocyte monolayers. In FBS‐containing media, the wound essentially closed within 48 hr (Figure 3a; Supporting Information Movie S1). This process was drastically slowed down by PSAP depletion. About 100 nM TX14(A) almost completely compensated for the loss of PSAP (Figure 3a,c; Supporting Information Movies S2 and S3). Importantly, knock‐down of GPR37L1/GPR37 in astrocytes blocked the ability of TX14(A) to facilitate wound closure while the control vector was ineffective (Figure 3c, Supporting Information Movies S4 and S5). Astrocytes divide in culture, albeit very slowly, but neither depletion of PSAP nor addition of TX14(A) affected the number of cells in cultures (Figure 3d) nor the number of newly divided cells based on BrdU staining (Figure 3e). Therefore, the effects of PSAP and TX14(A) in the scratch assay are due to their effect on the motility of astrocytes.

**Figure 4:**
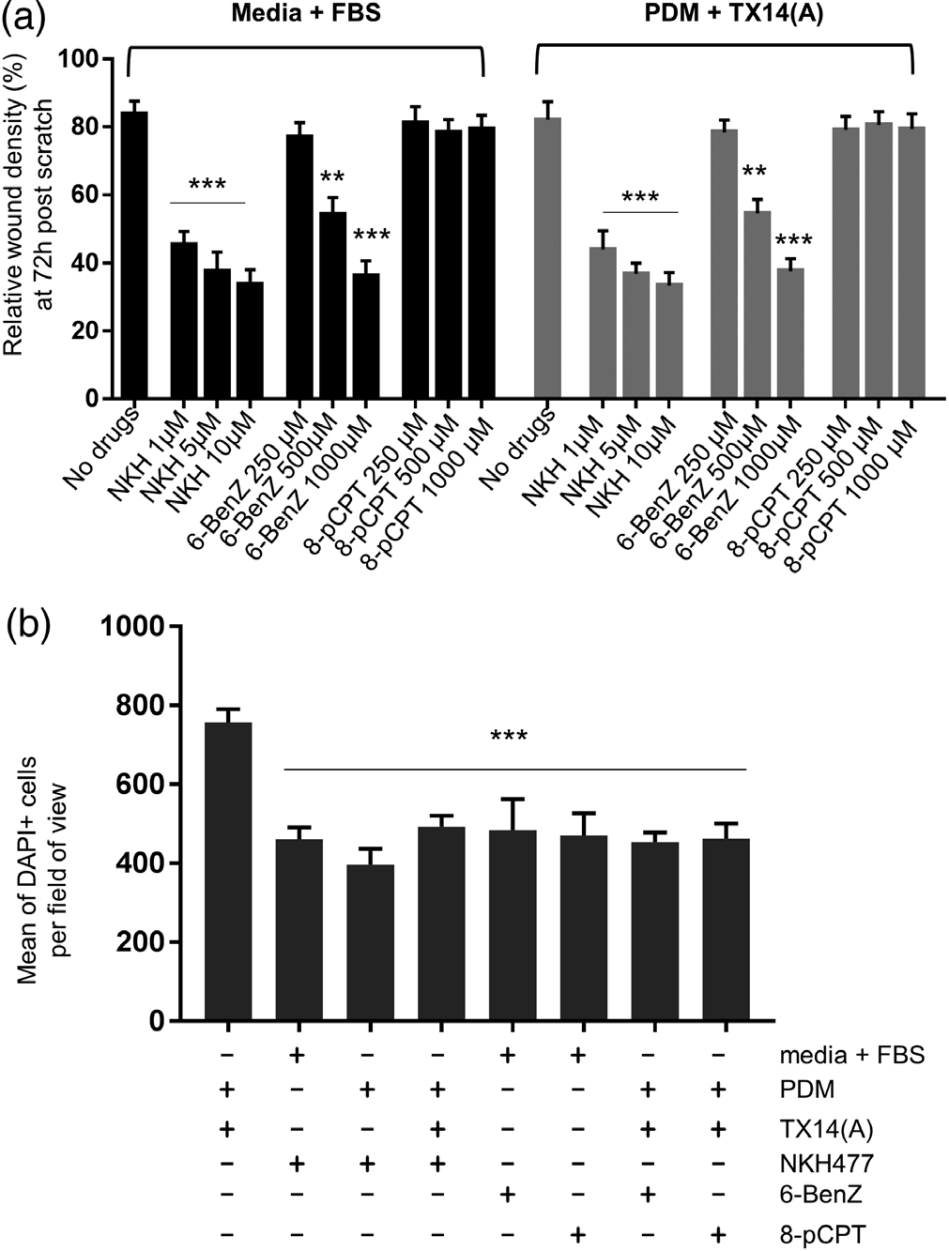
cAMP in astrocytes affects wound closure in the scratch assay. (a) A scratch wound was created in astrocyte monolayers cultured in FBS‐containing media or PDM supplemented with 100 nM TX14(A). Drugs were added and dynamics of the wound closure was monitored for 72 hr (*n* = 5, triplicates). (b) DAPI staining of astrocytes shows that NKH477 (10 μM), 6‐BenZ‐cAMP (500 μM), and 8‐pCPT‐2′‐O‐Me‐cAMP (1,000 μM) significantly decreased cell numbers as compared to control (*n* = 4, triplicates). In both cases, one‐way ANOVA with Dunnett's post hoc analysis. **p < .01, ***p < .001 vs. control [Color figure can be viewed at wileyonlinelibrary.com]

Stimulation of adenylate cyclase with NKH477 (1–10 μM) greatly slowed down wound closure, as did the cAMP analogue 6‐benz‐cAMP (250–1,000 μM), a selective PKA activator, in a concentration‐dependent manner (Figure 4a). The cAMP analogue 8‐pCPT‐2′‐O‐Me‐cAMP (250–1,000 μM) which specifically activates EPAC (cAMP‐GEF) had no effect (Figure 4a). Interestingly, nonselective stimulation of cAMP production by NKH477 as well as stimulation of PKA by 6‐BenZ‐cAMP and of EPAC by 8‐pCPT‐2′‐O‐Me‐cAMP reduced proliferative activity of astrocytes (Figures 3d and 4b). As all three cAMP raising drugs had a similar effect on cell numbers but different effects on wound closure dynamics, there appears to be no direct relationship between these two effects of cAMP. Taken together, the data indicate that the key mechanism of wound closure which is regulated via the GPR37L1/GPR37 axis is the lateral movement of astrocytes, rather than their division.

### 3.3 PSAP and prosaptide protect astrocytes from oxidative stress damage via GPR37L1/GPR37

**Figure 5:**
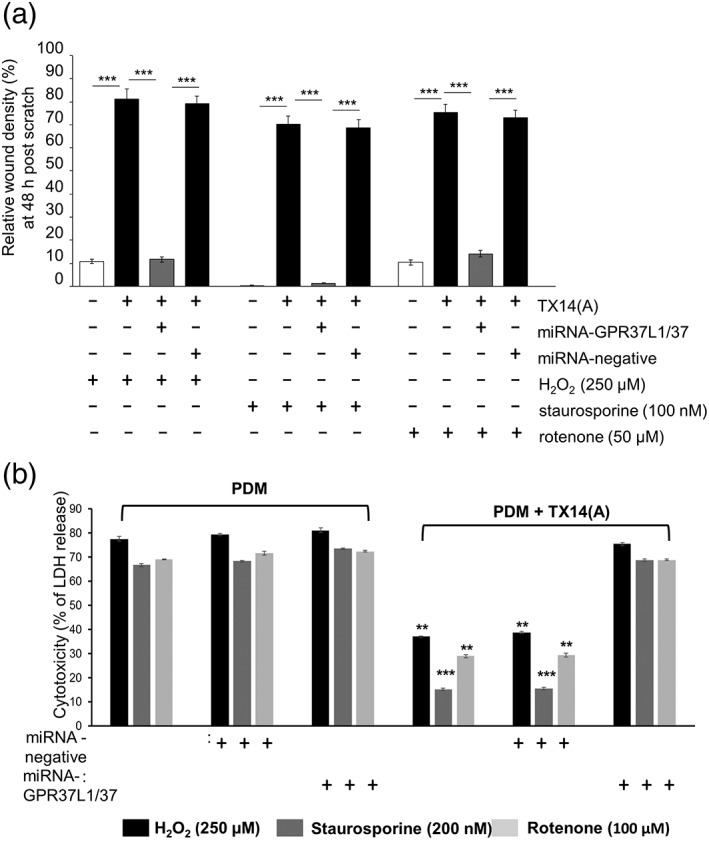
TX14(A) acts via GPR37L1 and GPR37 to protect primary astrocytes against toxicity induced by H_2_O_2_, staurosporine or rotenone. (a) Pre‐exposure to stressors (5 hr) drastically reduce relative wound density recorded at 48 hr in PDM. TX14(A) (100 nM) rescued astrocytes bringing wound density close to normal (compare to Figure 3). GPR37L1/GPR37 knock‐down prevented the protective effect of TX14(A), while the control vector had no effect (*n* = 6, triplicates, ***p < .001 vs. indicated group). (b) LDH release was used as a measure of cytotoxicity, 24 hr after exposure of astrocytes to oxidative stress. In PDM, manipulation of GPR37L1/GPR37 had no effect. TX14(A) (100 nM) protected them from damage but only when they were expressing GPR37L1/GPR37, (*n* = 6, triplicates, **p < .01, ***p < .001 vs. control group, for example, PDM groups). One‐way ANOVA with Bonferroni's post hoc analysis [Color figure can be viewed at wileyonlinelibrary.com]

Exposure of astrocytes to H_2_O_2_, rotenone, or staurosporine drastically inhibited their ability to close the wound in PSAP‐depleted media but TX14(A) rescued the stressed astrocytes' wound closure capacity (Figure 5a). This effect of TX14(A) was eliminated by the knock‐down of GPR37L1/GPR37 in astrocytes, while the control knock‐down vector was ineffective (Figure 5a).

It has been previously reported that the death of cortical astrocytes triggered by H_2_O_2_ can be reduced by TX14(A) (Meyer et al., 2013). We adjusted concentrations and exposure time of H_2_O_2_, rotenone, and staurosporine to evoke a comparable degree of cytotoxicity as assessed by LDH release assay (Figure 5b). In PSAP‐depleted media, neither GPR37L1/GPR37 knock‐down, nor the negative control vector, changed the cytotoxic impact of stressors. TX14(A) strongly reduced cytotoxicity which was particularly prominent in the case of staurosporine, and this was prevented by GPR37L1/GPR37 knock‐down (Figure 5b).

### 3.4 Astrocytic GPR37L1/GPR37 signaling contributes to the protection of the cortical neurons from damage by oxidative stressors

**Figure 6:**
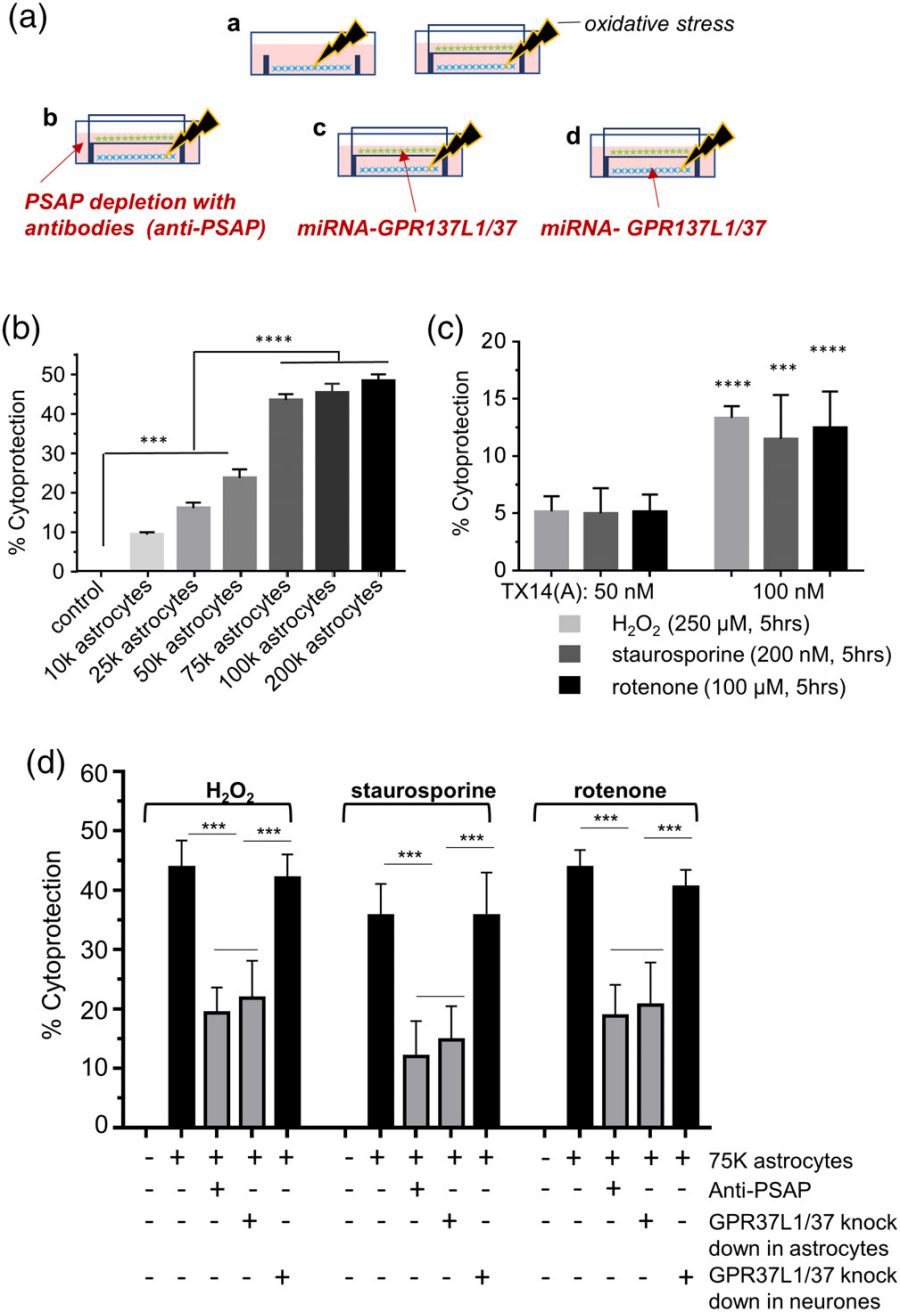
Co‐cultured astrocytes protect cortical neurons against oxidative toxicity partially through GPR37L1/GPR37 signaling in astrocytes. (a) Experimental design: (A—stressed neurons (blue) in absence or presence of astrocytes (green) on a culture insert; B—depletion of PSAP from the media with anti‐PSAP antibodies; C—GPR37L1 and GPR37 knock‐down in astrocytes co‐cultured with neurons; D—GPR37L1 and GPR37 knock‐down in neurons in the co‐culture system. Neurons were treated with H_2_O_2_ (250 μM) for 1 hr, or staurosporine (100 nM) or rotenone (50 μM) for 2 hr. Stressors were then removed and inserts with astrocytes were introduced. LDH assay was carried out 24 hr later. (b) Astrocytes protect cortical neurons against H_2_O_2_‐induced stress, the effect saturates at ~75 k astrocytes per co‐culture (*n* = 5, triplicates, ***p < .001 vs. control [no astrocytes insert], ****p < .0001 vs. groups with less than 50 k astrocytes, one‐way ANOVA with Tukey's post hoc analysis). (c) TX14(A) has a weak protective effect on neurons against oxidative stress (*n* = 6, triplicates, ***p < .001, ****p < .0001 vs. 50 nM TX14(A) group (effect of 50 nM is not significant, one‐way ANOVA with Bonferoni's post hoc analysis). D: PSAP depletion or GPR37L1/GPR37 knock‐down selectively in astrocytes significantly attenuates the protective effect of astrocytes on neurons pre‐exposed to oxidative stressors (*n* = 6, triplicates, ***p < .001 vs. indicated group, one‐way ANOVA with Turkey's post hoc analysis) [Color figure can be viewed at wileyonlinelibrary.com]

Neuronal cultures were subjected to the same oxidative stressors as above and conditions were adjusted to trigger a comparable degree of damage, based on LDH release. After removal of the stressors, astrocytes were introduced into the wells on elevated membranes, thereby preventing direct cell–cell contact (Figure 6a). Astrocytes exerted a strong protective effect on the neurons which was directly proportional to the quantity of astrocytes (Figure 6b; Supporting Information Figure S8). About 75 k astrocytes per well provided a near‐maximum neuroprotective effect and this astrocyte density was used for further experiments.

TX14(A) applied directly to damaged cultured neurons at 100 nM, provided a slight but significant protection against the stressors (Figure 6c). This indicates that either neuronal GPR37L1/GPR37 receptors, or the few astrocytes remaining in the neuronal cultures, could be contributing to the neuroprotective actions of PSAP.

Figure 6d demonstrates that, for each of the three stressors, co‐culturing with astrocytes reduced LDH release by ~35–45%. Importantly, the media used in these experiments was FBS‐free and contained no added PSAP. However, when anti‐PSAP antibodies were added, the cytoprotective effect of astrocytes was dramatically reduced (Figure 6d). This strongly suggests that endogenous PSAP (or Sap C) in the co‐culture system is important for the cytoprotective effect of astrocytes. We were able to immunodetect directly the increase in PSAP in co‐culture media in response to H_2_O_2_ stress applied to neurons (Supporting Information Figure S9), although the cellular source of this the stress‐induced PSAP surge is currently unknown. Knock‐down of GPR37L1/GPR37 selectively in astrocytes limited astrocyte‐mediated neuroprotection to a degree comparable to PSAP depletion (Figure 6d). Interestingly, application of the same knock‐down AVV to neurons had no effect. These results are consistent with the idea that stress‐induced PSAP release and astrocytic GPR37L1/GPR37 signaling are critical for PSAP action.

## 4 DISCUSSION

The key message of this study is that the original coupling of PSAP and its prosaptide fragments to GPR37L1 and GPR37 suggested by Meyer et al. (2013) was correct. This implies that we can expect small molecules based around the structure of TX14(A) be astro‐ and neuroprotective.

### 4.1 Neuroprotection by PSAP

The first cells used to demonstrate a protective potential of PSAP were mouse neuroblastoma NS20Y and human neuroblastoma SK‐N‐MC cells (O'Brien et al., 1994). Interestingly, the neuroblastoma lines closely related to SK‐N‐MC express substantial levels of GPR37 (Harenza et al., 2017). Published transcriptomes of astrocytes (Anderson et al., 2016; Chai et al., 2017; Zhang et al., 2014; Zhang et al., 2016) and our own data (Supporting Information Figures S2 and S3) unequivocally demonstrate that astrocytes of various parts of the central nervous system express high levels of GPR37L1 while the level of GPR37 is generally much lower. Cytoprotective and “trophic” effects of PSAP and its fragments were found in diverse models and species. Prosaptides improved the outcome of sciatic nerve damage in guinea pigs (Kotani et al., 1996), alleviated the ischemia‐induced memory deficits in gerbils (Kotani et al., 1996), reduced neuropathy in diabetic rats (Calcutt et al., 1999), and the behavioral and anatomical detriments caused by brain wound insult in rats (Hozumi et al., 1999). A stabilized TX14(A)‐like peptide, retro–inverso prosaptide D5, was neuroprotective in rats (Lu, Otero, Hiraiwa, & O'Brien, 2000) and ameliorated hyperalgesia in a model of neuropathic pain (Yan, Otero, Hiraiwa, & O'Brien, 2000). The neuroprotective effects of TX14(A) were confirmed by another group (Jolivalt, Dacunha, Esch, & Calcutt, 2008; Jolivalt, Ramos, Herbetsson, Esch, & Calcutt, 2006; Sun et al., 2002). An 18‐amino acid long prosaptide was also protective in a model of dopaminergic neuron damage (Gao et al., 2013; Sun et al., 2002). Therefore, there is solid evidence for the neuroprotective potential of this pathway.

### 4.2 PSAP‐GPR37L1/GPR37 pairing

All these effects obviously called for the development of a neuroprotective therapy, but this opportunity could not be realized in the absence of cognate receptors. The study by Meyer et al. (2013) strongly suggested that these are the orphan receptors GPR37L1 and GPR37. Some of the effects were demonstrated in HEK293 cells, but the most striking protective effect of TX14(A) was observed in cultured astrocytes. The findings of Meyer et al. (2013) were later criticized, the key concerns being the high concentration of TX14(A) used, the small magnitude of the G_i_‐mediated inhibitory effect on cAMP concentration and the failure to detect TX14(A) agonism in a β‐arrestin‐based DiscoverX assay (Smith, 2015; Southern et al., 2013). Other recent studies reported high constitutive activity of GPR37L1 and GPR37 and lack of a TX14(A) effect in either HEK293 cells or yeast (Coleman et al., 2016; Giddens et al., 2017; Ngo et al., 2017; Southern et al., 2013).

Our findings, however, are consistent with the conclusions drawn by (Meyer et al., 2013). The presence of PSAP in the media or addition of TX14(A) had a powerful effect on astrocytic motility and protected them against oxidative stressors. In all cases, this action could be completely prevented by knocking down GPR37L1/GPR37 (Figures 3a,c and 5). Moreover, when astrocytes were used to rescue neurons subjected to oxidative stress, removal of GPR37L1/GPR37 only from astrocytes was sufficient to significantly weaken their neuroprotective capacity and block the protective action of TX14(A) (Figure 6d). TX14(A) at a fairly high concentration (100 nM) had a weak direct protective effect on stressed neurons which is unlikely to make a major contribution to the neuroprotection seen in the presence of astrocytes (Figure 6c). At least, application of the knock‐down strategy to neurons was without an obvious effect (Figure 6d).

**Figure 7:**
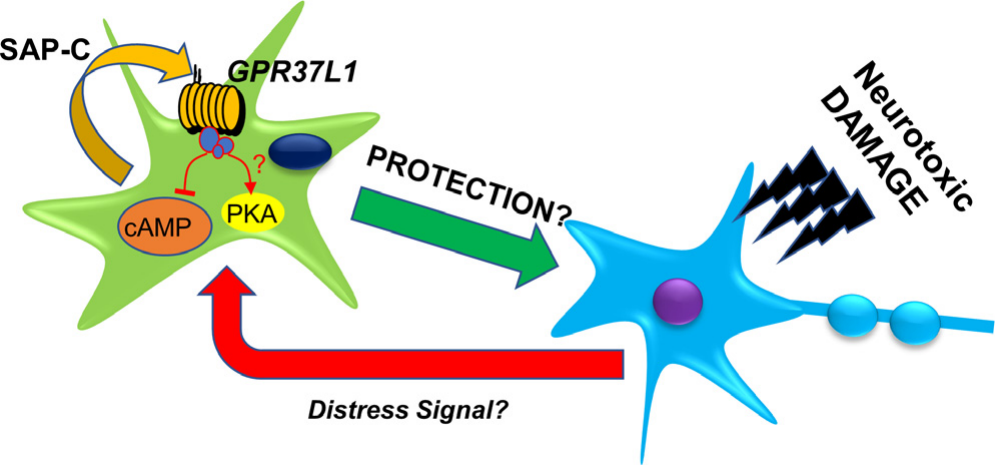
Working model of the neuroprotective role of astrocytic GPR37L1/GPR37 based on the evidence presented in this study. Damaged neurons release diffusible “SOS” factor(s) which trigger release of PSAP. PSAP acts on GPR37L1 on astrocytes and activates release of diffusible neuroprotective factor(s) [Color figure can be viewed at wileyonlinelibrary.com]

Therefore, by several approaches, we demonstrate that the effects of PSAP and TX14(A) on astrocytes are invariably dependent on GPR37L1/GPR37. The partial protection provided to injured neurons by co‐cultured astrocytes to some extent also depends on this signaling pathway. Given that only removal of GPR37L1/GPR37 from astrocytes, but not neurons interfered with neuroprotection in this paradigm, the likeliest scenario is that PSAP acts as an autocrine signal on the receptors located on the astrocytes to recruit additional, unidentified, neuroprotective molecules (Figure 7).

Previous studies in mice lacking either of the two receptors demonstrated various, albeit relatively mild, phenotypes but, to the best of our knowledge, the possibility of compensation by the remaining receptor in a single knockout scenario has never been explored. This could be the reason why the pro‐seizure phenotype of double GPR37L1/GPR37 knockout mice is so severe (Giddens et al., 2017). Recent demonstration of a lethal neurological phenotype in humans with a point mutation in GPR37L1 (Giddens et al., 2017) suggests that GPR37L1 is potentially indispensable for the health of the human brain. A drastic increase in neuronal loss after an ischemic stroke in GPR37L1 knockout mice (Jolly et al., 2017) further reinforces the importance of these receptors.

### 4.3 Coupling

The first study to indicate that a G_i_‐coupled receptor may mediate the action of PSAP found that pretreatment with PTX inhibited agonist‐stimulated binding of [^35^S]‐GTPγS (Hiraiwa et al., 1997). Strikingly, it also demonstrated that Sap C interacts with a receptor of ~54 kDa, corresponding almost exactly to the molecular mass of GPR37L1 (Giddens et al., 2017). In our study in astrocytes, TX14(A) inhibited cAMP production by ~40% from an elevated level set by the forskolin analogue (Glosensor assay). The effect of TX14(A) was PTX sensitive and the IC_50_ compares with that of numerous other GPCR agonists. Removal of GPR37L1 and GPR37 completely prevented the effect of TX14(A). These results confirm the original report (Meyer et al., 2013) that GPR37L1/GPR37 are G_i_‐coupled receptors. Coupling to other G‐proteins needs to be investigated further.

In the wound scratch assay, PSAP and TX14(A) were permissive to the spread of astrocytes into the barren area. This was due to an effect on lateral motility (Figure 3d,e). Again, this effect was completely prevented by knock‐down of the two receptors (Figure 3c). Interestingly, stimulants of the cAMP pathway, the adenylyl cyclase activator NKH477, and two pathway‐biased agonists, 6‐BenZ‐cAMP, and 8‐pCPT‐2′‐O‐Me‐cAMP, reduced the speed of division of cultured astrocytes (Figure 4b) but only 6‐BenZ‐cAMP which predominantly activates PKA (Bos, 2003) concentration‐dependently suppressed spread of the astrocytes into the wounded area (Figure 4a). Therefore, the motility of astrocytes is regulated by PKA, rather than EPAC‐regulated proteins. TX14(A) did not interfere with the reduction in mitotic activity by either of the cAMP analogs (Figure 4b). The mechanism by which PKA regulates astrocyte motility is currently unknown.

### 4.4 Controversy related to PSAP‐GPR37L1/GPR37 pairing

Neither HEK293 cells nor yeast used in the recent conflicting studies (Coleman et al., 2016; Giddens et al., 2017; Ngo et al., 2017) express GPR37L1 or GPR37 natively, nor were they ever demonstrated to be responsive to PSAP or prosaptides. These cells might not necessarily recapitulate the intracellular environment of astrocytes and neurons which are the natural habitats of GPR37L1 and GPR37. Our experiments using PRESTO‐Tango confirmed that neither GPR37L1 nor GPR37 respond to TX14(A) in HEK293 cells (Figure 2).

Serum‐containing media can mask the effects of GPR37L1/GPR37 ligands because it contains considerable levels of PSAP. Most likely astrocytes, neurons or both can secrete some PSAP in vivo. This was visible in the “rescue” experiments where astrocytes reduced the damage caused to neurons by added stressors. In these experiments, anti‐PSAP antibodies reduced neuroprotection even though the media was nominally devoid of PSAP. In the presence of stressed neurons, PSAP was greatly upregulated and easily detected in the media by immunoblotting. Therefore, to fully reveal the agonist activity of TX14(A), it is important to ensure that the receptors are not persistently exposed to endogenous or media‐derived PSAP.

Taken together, our results demonstrate that prosaptide TX14(A) (and, by extension, PSAP) are the natural ligands of GPR37L1/GPR37 and confirm that in their native environment, the astrocyte, these receptors couple to the G_i_ cascade as originally reported (Meyer et al., 2013). Their native signaling is, however, lacking or suppressed in cell lines (such as transiently transfected HEK293 cells) used in β‐arresting‐based screening assays, indicating that for correct coupling GPR37L1 and GPR37 may require as yet unknown intracellular partners present in astrocytes. This would not be a unique situation since some other receptors, such as CGRP receptor, are known to require co‐expression of receptor‐associated proteins. Of note, astrocytes express very high levels of syntenin‐1, which is important for trafficking of GPR37 (Dunham, Meyer, Garcia, & Hall, 2009). It is conceivable that its role includes more than just trafficking. Given the powerful glio‐protection and neuroprotection mediated by these receptors and since GPCRs are the most “druggable” class of proteins currently known, GPR37L1 (and GPR37, if they can be separated pharmacologically) become highly valuable targets for development of novel neuroprotective therapies.

## Supporting information


**Figure S1:** Alignment of prosaptide TX14 against PSAP of various species

**Figure S2:** Expression levels of several GPR37L1, GPR37 assessed by next generation sequencing

**Figure S3:** Expression levels of GPR37L1 and GPR37 in cultured and acutely isolated rat astrocytes assessed by Real Time PCR

**Figure S4:** Immunodetection of PSAP contained in fetal calf serum and cell culture media

**Figure S5:** GPR37L1 and GPR37 knockdown efficiency verified by Real Time PCR

**Figure S6:** Astrocytes and neurons transduced with AVV‐miRNA‐GPR37L1/37

**Figure S7:** Response of Adra 1a receptor to norepinephrine detected by PRESTO‐TANGO system

**Figure S8:** Astrocytes protect cortical neurons against oxidative stress induced by rotenone and staurosporine in a “number”‐dependent manner.

**Figure S9:** PSAP is produced by astrocytes and neurons.

**Movie S1:** Wound closure dynamics in full media

**Movie S2:** Wound closure dynamics in PSAP‐depleted media (PDM)

**Movie S3:** Wound closure dynamics in PSAP‐depleted media supplemented with TX14(A) 100 nM

**Movie S4:** Wound closure dynamics in PSAP‐depleted media supplemented with TX14(A) 100 nM in astrocytes where GPR37L1 and GPR37 were knocked down

**Movie S5:** Wound closure dynamics in PSAP‐depleted media supplemented with TX14(A) 100 nM in astrocytes treated with vectors expressing control (negative) knock‐down cassette
