# Supplementary figures and images for: Glio‐ and neuro‐protection by prosaposin is mediated by orphan G‐protein coupled receptors GPR37L1 and GPR37
Source: Glia. 2018 Sep 27;66(11):2414–26. doi: 10.1002/glia.23480 (PMC6492175; doi:10.1002/glia.23480)

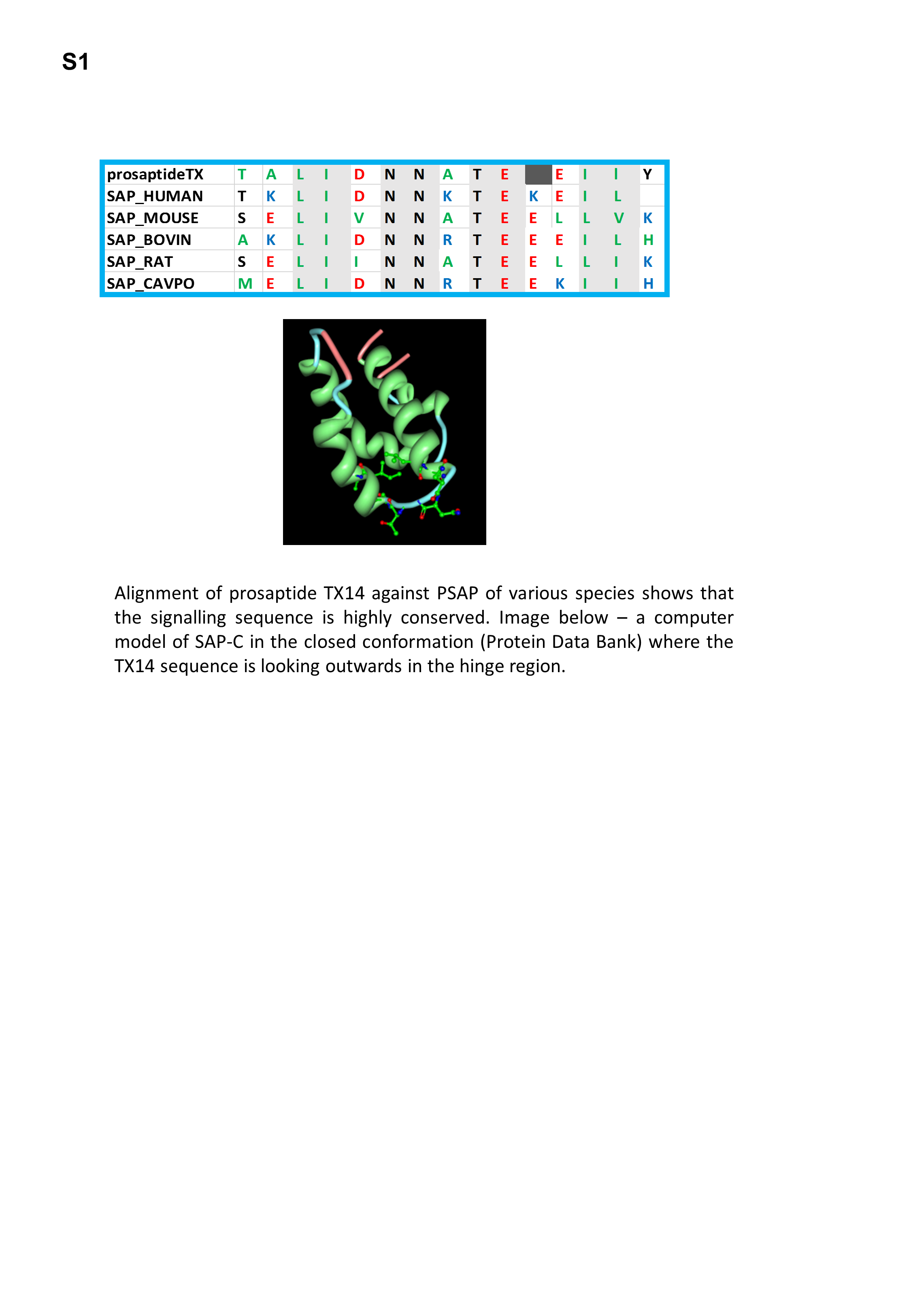

Supplement: Supplementary file 1 — Figure S1: Alignment of prosaptide TX14 against PSAP of various species [file GLIA-66-2414-s001.TIF]

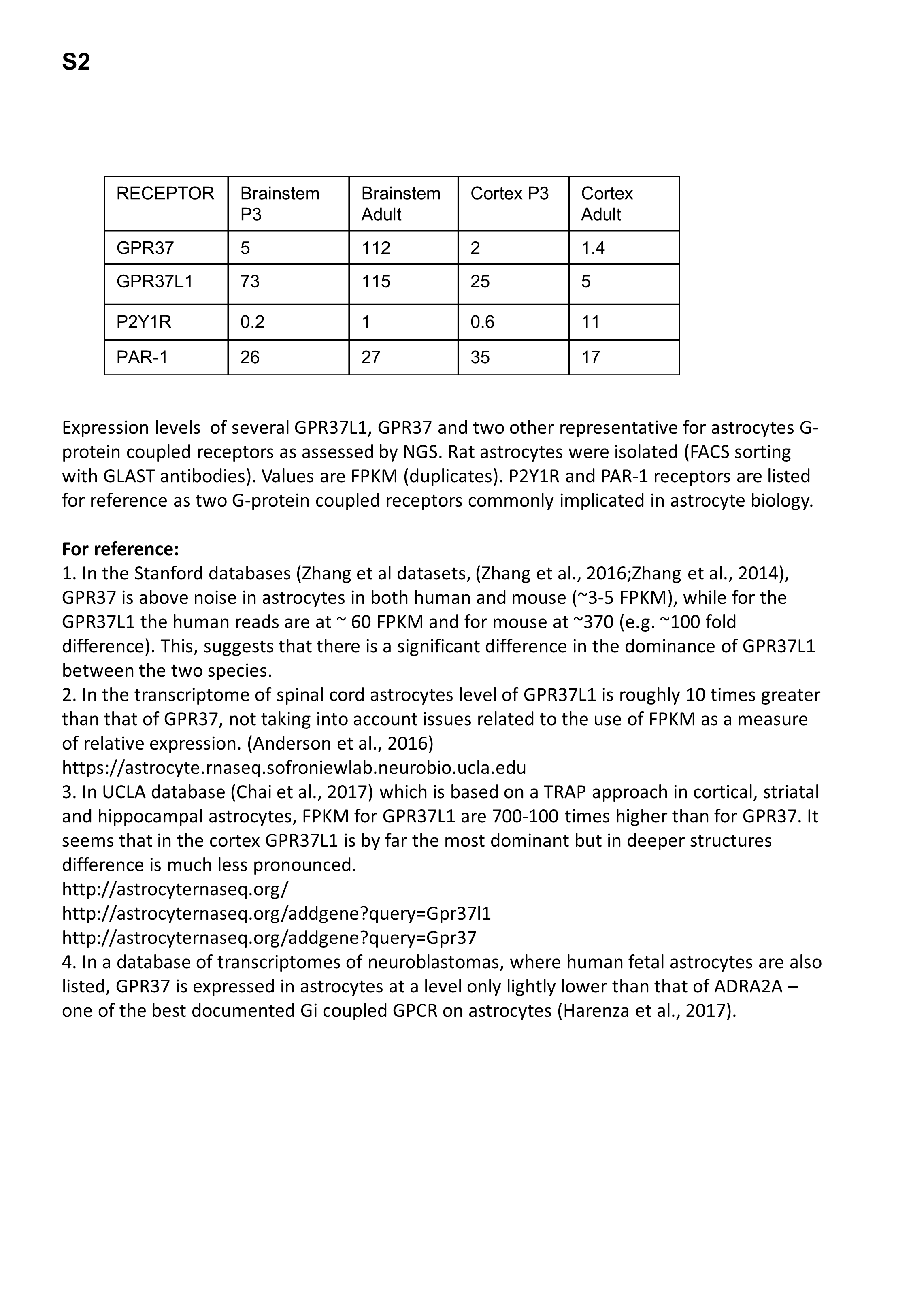

Supplement: Supplementary file 2 — Figure S2: Expression levels of several GPR37L1, GPR37 assessed by next generation sequencing [file GLIA-66-2414-s002.TIF]

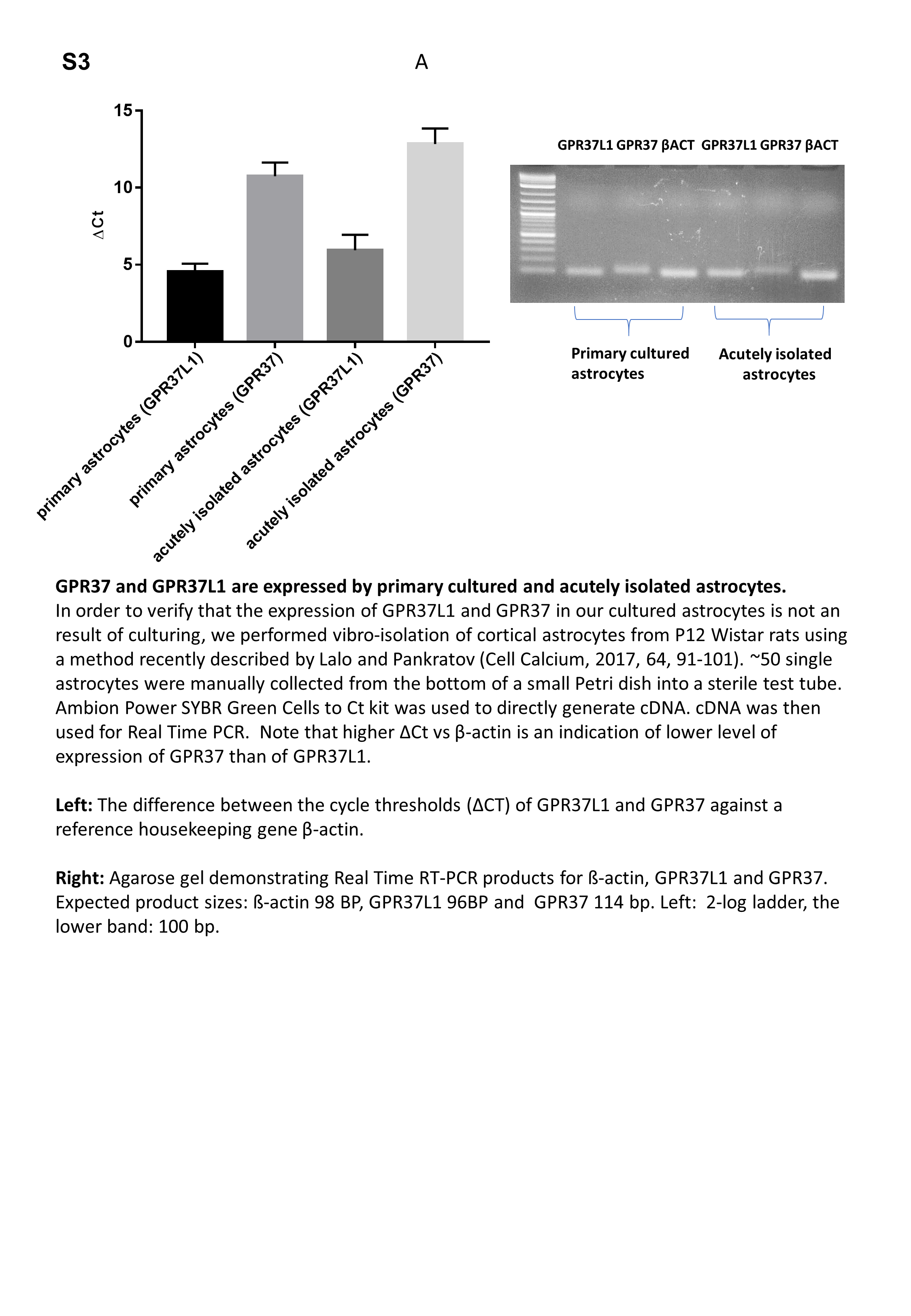

Supplement: Supplementary file 3 — Figure S3: Expression levels of GPR37L1 and GPR37 in cultured and acutely isolated rat astrocytes assessed by Real Time PCR [file GLIA-66-2414-s003.TIF]

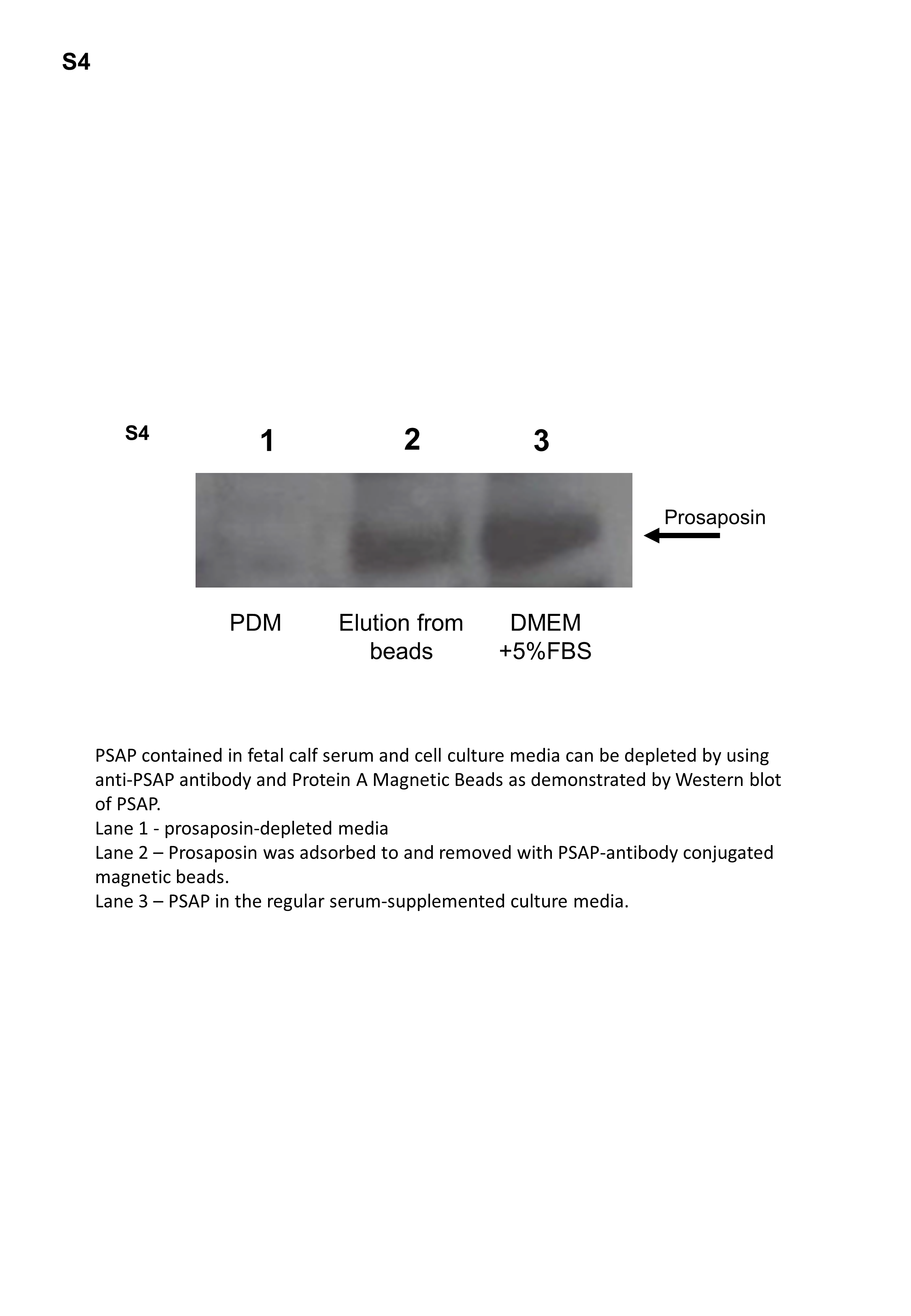

Supplement: Supplementary file 4 — Figure S4: Immunodetection of PSAP contained in fetal calf serum and cell culture media [file GLIA-66-2414-s004.TIF]

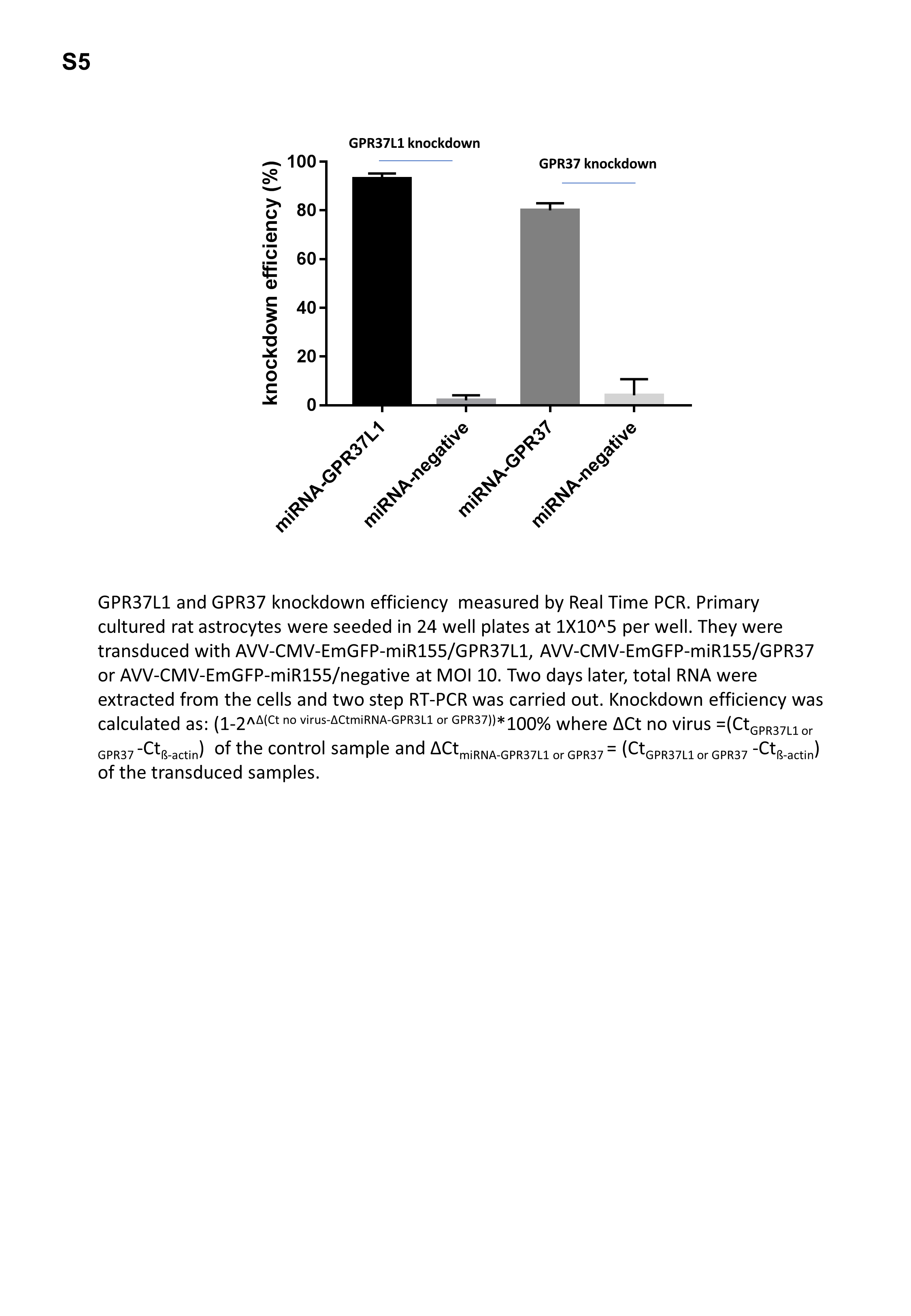

Supplement: Supplementary file 5 — Figure S5: GPR37L1 and GPR37 knockdown efficiency verified by Real Time PCR [file GLIA-66-2414-s005.TIF]

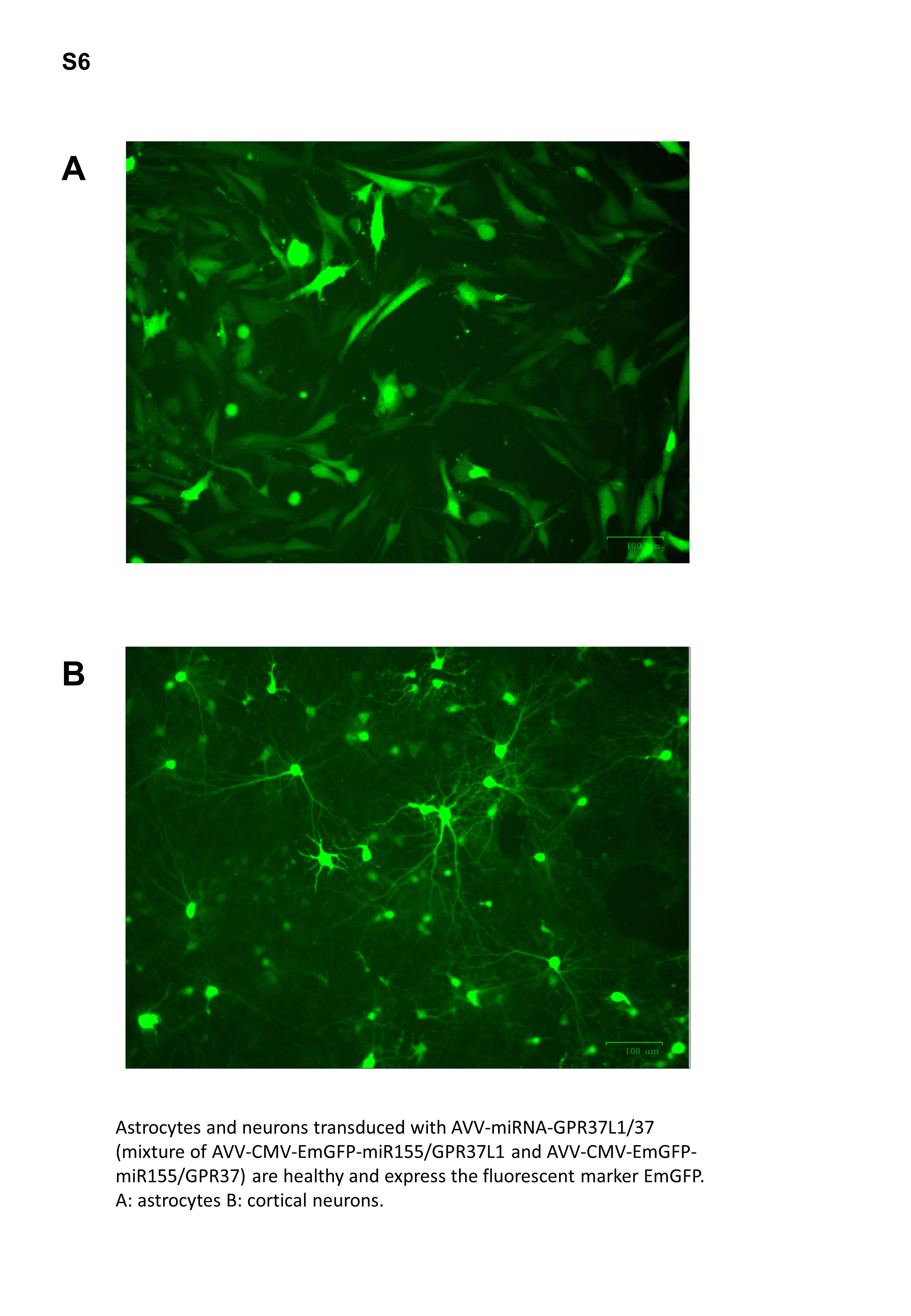

Supplement: Supplementary file 6 — Figure S6: Astrocytes and neurons transduced with AVV‐miRNA‐GPR37L1/37 [file GLIA-66-2414-s006.TIF]

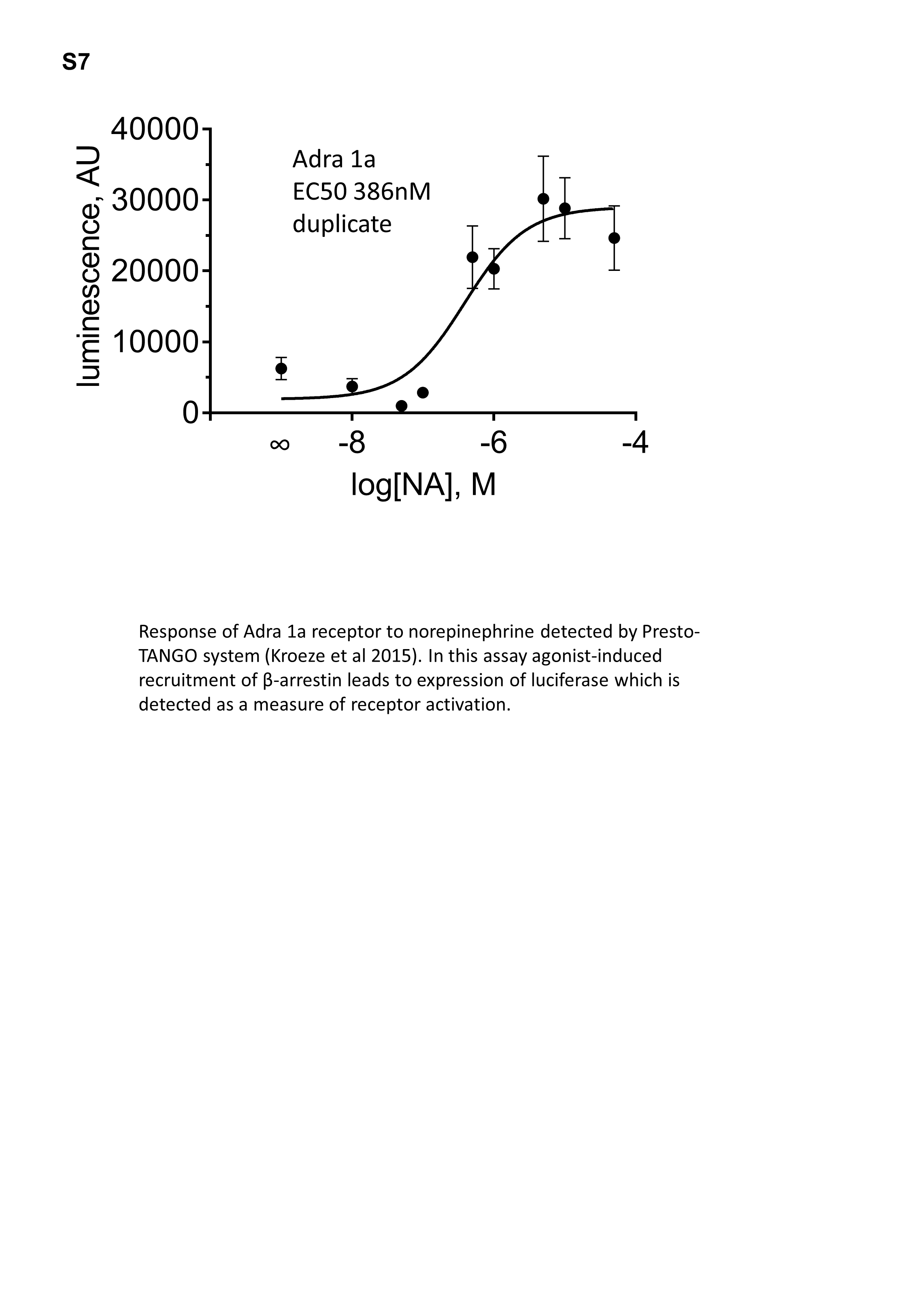

Supplement: Supplementary file 7 — Figure S7: Response of Adra 1a receptor to norepinephrine detected by PRESTO‐TANGO system [file GLIA-66-2414-s007.TIF]

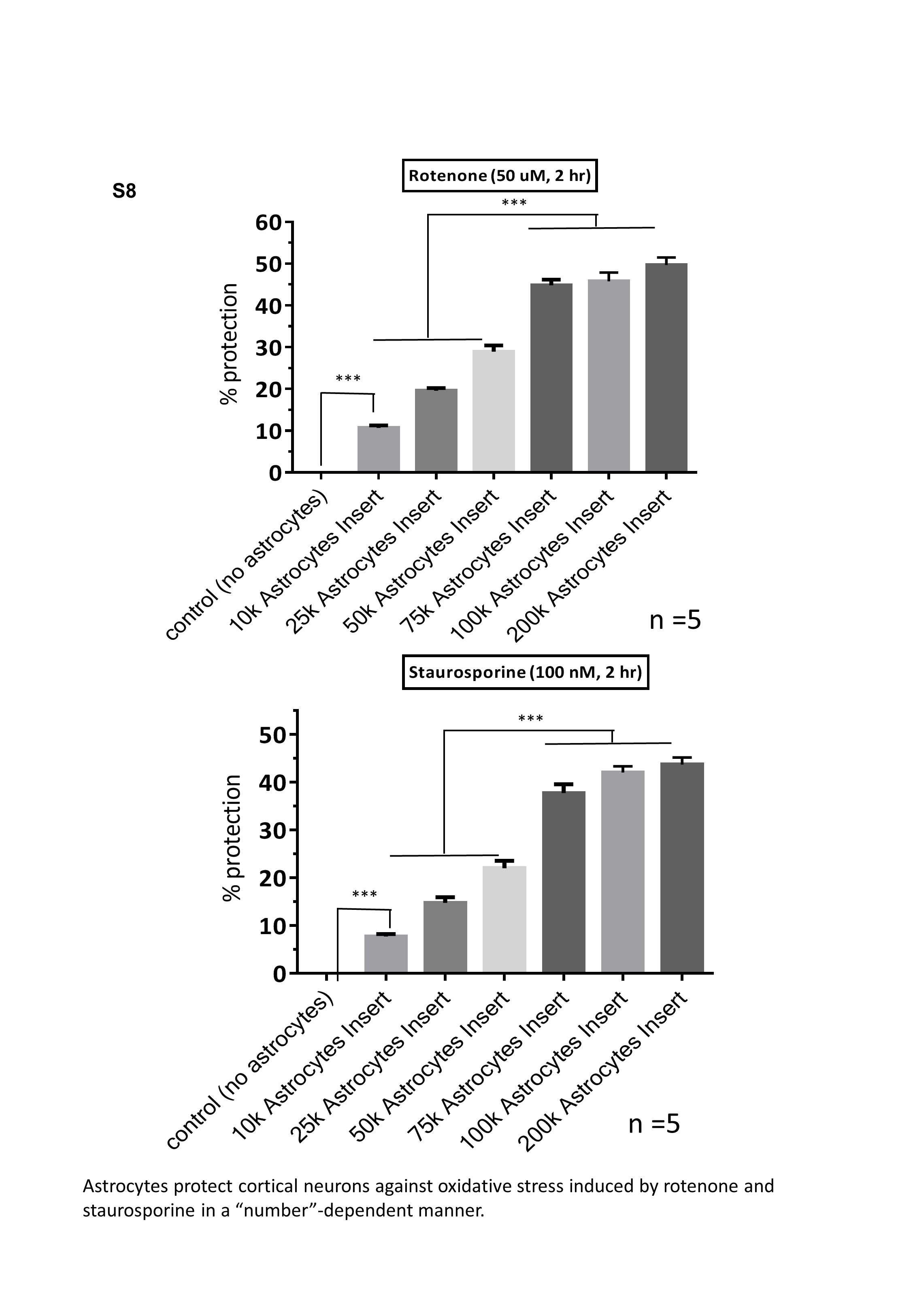

Supplement: Supplementary file 8 — Figure S8: Astrocytes protect cortical neurons against oxidative stress induced by rotenone and staurosporine in a “number”‐dependent manner. [file GLIA-66-2414-s008.TIF]

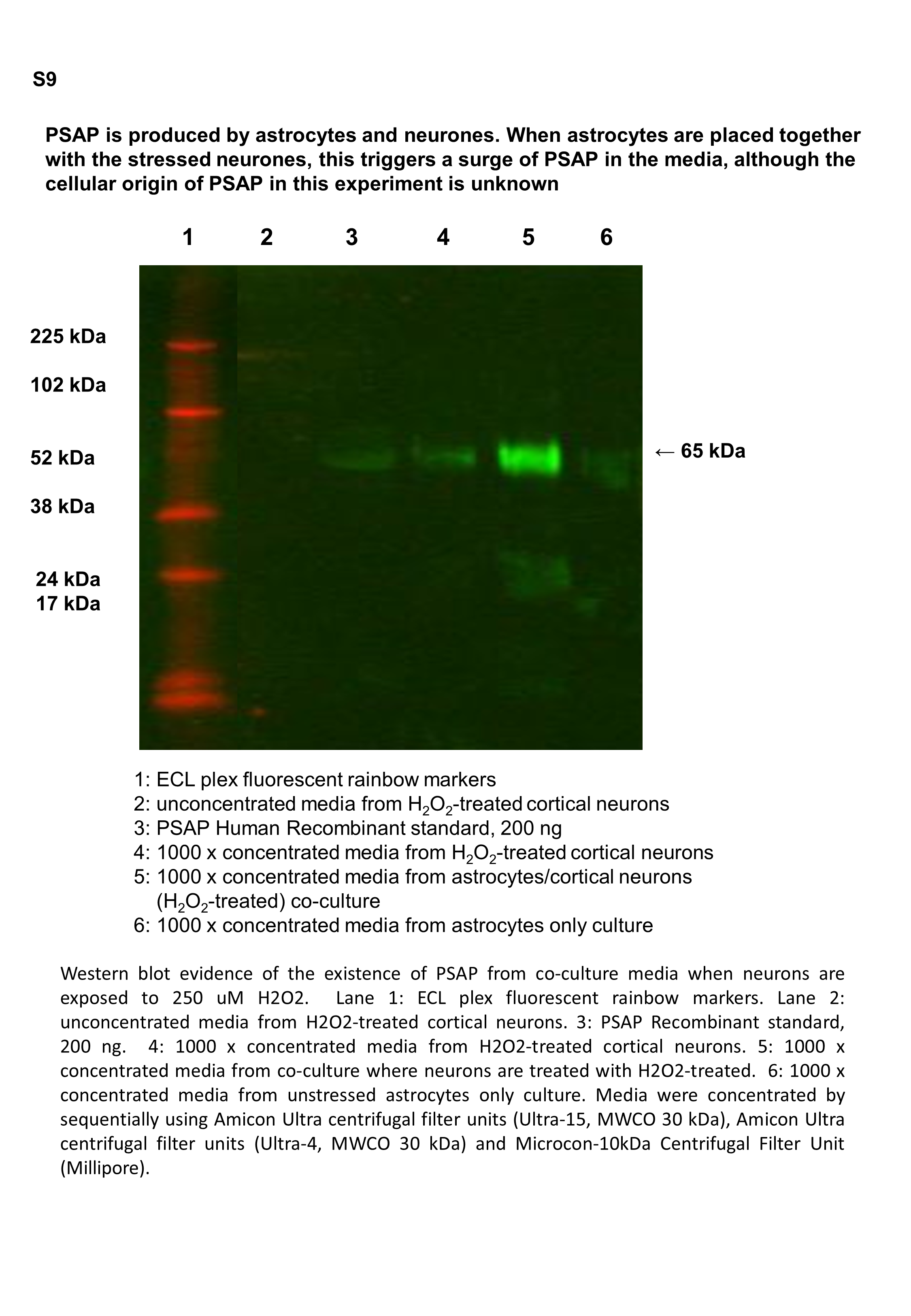

Supplement: Supplementary file 9 — Figure S9: PSAP is produced by astrocytes and neurons. [file GLIA-66-2414-s009.TIF]
